# Laying it on thick: Ecosystem effects of sediment placement on a microtidal Rhode Island salt marsh

**DOI:** 10.3389/fenvs.2022.939870

**Published:** 2022-09-06

**Authors:** Kenneth B. Raposa, Michael Bradley, Caitlin Chaffee, Nick Ernst, Wenley Ferguson, Thomas E. Kutcher, Richard A. McKinney, Kenneth M. Miller, Scott Rasmussen, Elizabeth Tymkiw, Cathleen Wigand

**Affiliations:** 1 RI Department of Environmental Management, Narragansett Bay National Estuarine Research Reserve, Prudence Island, RI, United States; 2 Department of Natural Resources Science, University of Rhode Island, Kingston, RI, United States; 3 U.S. Fish and Wildlife Service, Department of Interior, Rhode Island National Wildlife Refuge Complex, Charlestown, RI, United States; 4 Save The Bay, Providence, RI, United States; 5 Rhode Island Natural History Survey, Kingston, RI, United States; 6 Atlantic Coastal Environmental Sciences Division, U.S. Environmental Protection Agency, Narragansett, RI, United States; 7 General Dynamics Information Technology, Falls Church, VA, United States; 8 Northeast Coastal and Barrier Network, National Park Service, University of RI, Kingston, RI, United States; 9 Department of Entomology and Wildlife Ecology, University of Delaware, Newark, DE, United States

**Keywords:** coastal marsh, restoration, sediment placement, adaptive management, ecological monitoring, elevation, vegetation, ecosystem responses

## Abstract

Heightened recognition of impacts to coastal salt marshes from sea-level rise has led to expanding interest in using thin-layer sediment placement (TLP) as an adaptation tool to enhance future marsh resilience. Building on successes and lessons learned from the Gulf and southeast U.S. coasts, projects are now underway in other regions, including New England where the effects of TLP on marsh ecosystems and processes are less clear. In this study, we report on early responses of a drowning, microtidal Rhode Island marsh (Ninigret Marsh, Charlestown, RI) to the application of a thick (10–48 cm) application of sandy dredged material and complimentary extensive adaptive management to quickly build elevation capital and enhance declining high marsh plant species. Physical changes occurred quickly. Elevation capital, rates of marsh elevation gain, and soil drainage all increased, while surface inundation, die-off areas, and surface ponding were greatly reduced. Much of the marsh revegetated within a few years, exhibiting aspects of classic successional processes leading to new expansive areas of high marsh species, although low marsh *Spartina alterniflora* recovered more slowly. Faunal communities, including nekton and birds, were largely unaffected by sediment placement. Overall, sediment placement provided Ninigret Marsh with an estimated 67–320 years of ambient elevation gain, increasing its resilience and likely long-term persistence. Project stakeholders intentionally aimed for the upper end of high marsh plant elevation growth ranges to build elevation capital and minimize maintenance costs, which also resulted in new migration corridors, providing pathways for future marsh expansion.

## Introduction

Coastal salt marshes are transitional habitats that occur between uplands and estuaries and provide ecosystem services and societal benefits. They serve as habitat for fish, shellfish and wildlife, maintain water quality, and sequester carbon (Costanza et al., 1997; Barbier et al., 2011), and during the current period of human-accelerated environmental change (Crutzen, 2002), they are recognized for their capacity to buffer coasts from sea-level rise (SLR) and storm surges (Temmerman et al., 2004; Silliman et al., 2019). The trade-off to this buffer protection is that the marshes themselves are becoming more impacted in some regions as SLR accelerates.

In New England, where marshes are already impacted from legacy agriculture (e.g., ditching, embankments), reduced sediment supplies, and coastal development, accelerated SLR is now leading to rapid marsh change and degradation (Weston, 2014; Raposa et al., 2017a; Watson et al., 2017; Burdick et al., 2020). The highest rate of SLR in southern New England since 1850 occurred during the last decade and rates are predicted to further accelerate by 2100 (Gonneea et al., 2019; Oppenheimer et al., 2019). In Narragansett Bay, RI, the long-term rate of SLR (1930–2021) is 2.83 mm yr^−1^, but in the near-term (1989–2019, the most recent 30-year period for which data were available) this increased 68% to 4.78 mm yr^−1^ (Newport, RI NOAA tide station, ID 8452660; PSMSL, 2022). Predictably, recent historical analysis of photographic imagery indicates significant near-term marsh drowning and losses in the region (Watson et al., 2017). The rate of marsh change and loss has prompted resource managers and conservation groups to develop climate adaptation plans to enhance coastal marsh resilience against SLR (Kutcher and Chaffee, 2021). Possible adaptation actions include planning for upland marsh migration, installing living shorelines to minimize erosion and promote sediment deposition, and placing sediment on marsh platforms to build elevation capital (i.e., the relative elevation of the marsh platform within the intertidal zone; Cahoon and Guntenspergen, 2010) (Wigand et al., 2017). Marshes subject to accelerated SLR that are lower in the intertidal zone (low elevation capital) are in more imminent danger of drowning than marshes that are located higher (Cahoon and Guntenspergen, 2010).

One technique to build marsh resilience that is gaining in popularity is thin-layer sediment placement (TLP; Croft et al., 2006; VanZomeren et al., 2018; Thorne et al., 2019; Raposa et al., 2020). This approach mimics aspects of natural sediment deposition on marshes by strategically adding, or “placing,” sediment derived from a source away from the marsh (typically nearby dredged sediment) onto degraded, low-elevation marsh areas to increase elevation capital and extend the lifespan of the marsh. Thin sediment layers (often <10 cm) are typically added to avoid completely burying existing plants and allow some vegetation recovery to occur *via* regrowth of existing plants through the new sediment. A growing body of literature demonstrates beneficial results from TLP including enhanced elevation capital and vegetation biomass *via* reduced inundation, better drainage, and increased redox potential (Croft et al., 2006; Walters and Kirwan, 2016; Berkowitz and VanZomeren, 2020), although thickness and chemistry of added sediments can inhibit vegetation recovery (Puchkoff and Lawrence, 2022). Most studies evaluating TLP have been conducted in Gulf and southeast U.S. marshes, and many focused primarily on edaphic conditions and low marsh vegetation (Mendelssohn and Kuhn, 2003; Stagg and Mendelssohn, 2010; Tong et al., 2013). Additional large-scale case studies are needed from other regions, especially those undergoing rapid SLR acceleration, and a more comprehensive suite of parameters needs to be examined (Raposa et al., 2020). New England is experiencing very high rates of SLR (Sallenger et al., 2012) but very few TLP studies have been conducted in this region and recent published accounts describe results from natural deposition events (Moore et al., 2021) or smaller-scale organ experiments (Payne et al., 2021; Puchkoff and Lawrence, 2022).

Our study helps to fill this gap by describing physical and biological changes from one of the first large-scale sediment placement projects in New England where 26,000 cubic meters of dredged sandy sediments (10–48 cm thick) were added to a low-elevation, drowning marsh in a microtidal coastal lagoon in southern RI. We hereafter deviate from using the term “TLP” in favor of “sediment placement” due to the thickness of sediment added to the marsh in this study. Dredged sediments were a product of a coupled effort to dredge and deepen a channel within an adjacent breachway to improve navigation for recreational boaters. The project was conducted using a coordinated consortium of policy analysts, restoration practitioners, and coastal researchers representing local, state, and federal government agencies, nonprofits, and universities (Mulvaney et al., 2022).

The primary goals of the project were to restore high marsh species to portions of the marsh that had been degraded due to prolonged surface water ponding, and increase marsh resilience to future SLR using targeted sediment placement (Fuss & O’Neill, 2015). The overarching conceptual project objective was to test the efficacy of sediment placement coupled with subsequent adaptive management and monitoring as a practical and scientifically-sound approach for building marsh resilience to SLR. The specific experimental objective was to quantify ecological effects of sediment placement on a drowning marsh by monitoring key indicative parameters before and after sediment placement and at a nearby control marsh. We monitored hydrological, geophysical, and biological responses to the sediment placement including but not limited to: elevation, hydrology, salinity, soils, vegetation, nekton, and birds. It was expected that sediment placement would change the trajectory of the marsh towards a more stable and improved condition than the nearby drowning control marsh. Our study provides an example of how tidal marshes respond to this emerging climate adaptation technique (i.e., sediment placement), and results can help guide and improve monitoring and evaluation of future coastal marsh resilience projects.

## Materials and methods

### Study site

**FIGURE 1:**
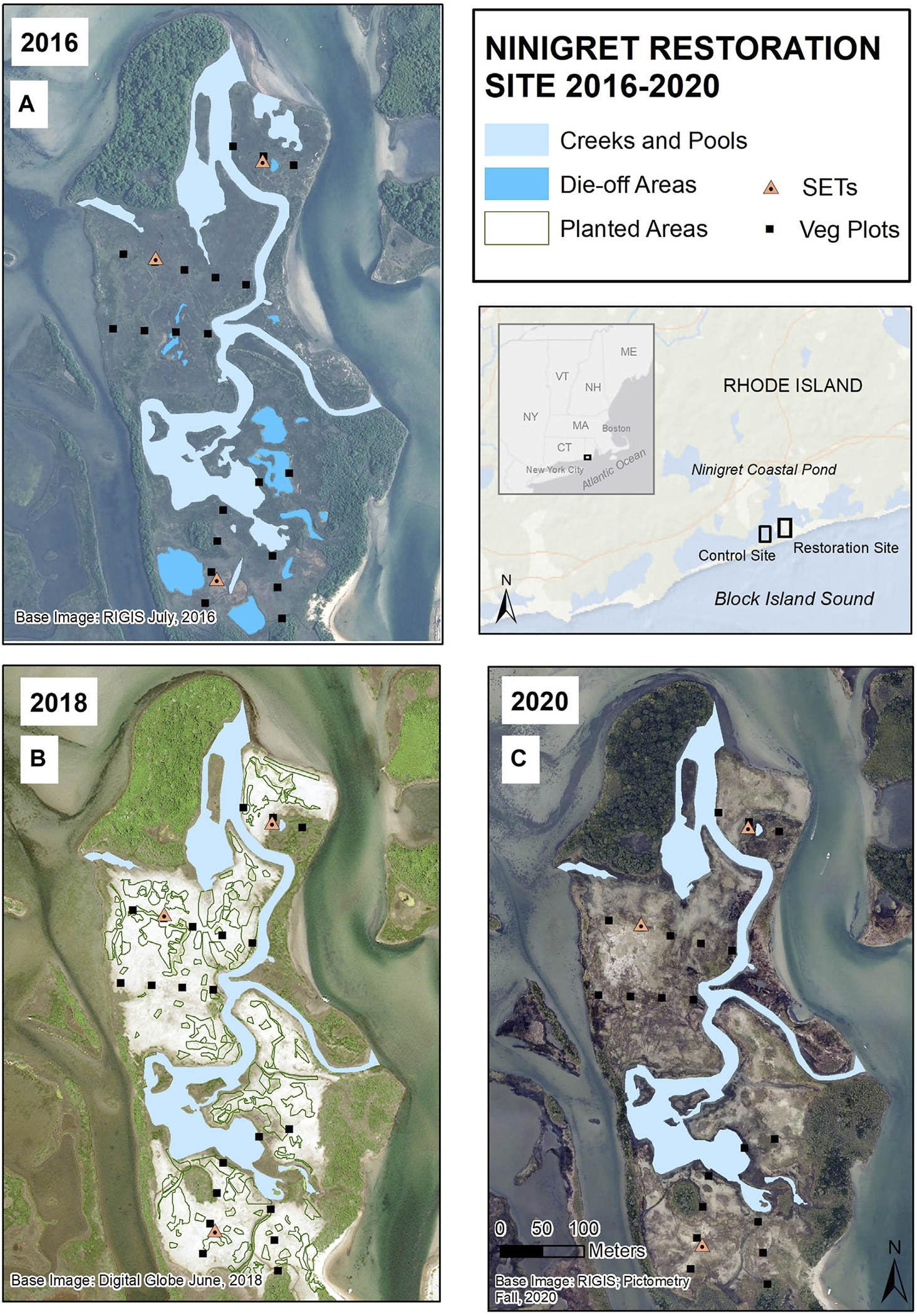
Locus maps showing the Ninigret sediment placement site in Ninigret Pond, Charlestown RI, and time-series aerial photo maps showing **(A)** initial degraded conditions in 2016, **(B)** expansive unvegetated areas in 2018 two summers after sediment placement (with polygons depicting the estimated outlines of planted areas) and **(C)** vegetation re-growth in 2020. Vegetation monitoring plots and surface elevation tables (SETs), none of which were located in planted areas, are shown on each map. Note that the 2016 and 2018 photos were taken in summer; the 2020 photo was taken in early fall.

The 15-ha study site is located within the coastal barrier complex along the southern edge of Ninigret Pond in Charlestown, RI (Figure 1). Ninigret Pond is a polyhaline, microtidal (<1 m), wind-driven system (Boothroyd et al., 1985) and at ~690 ha is one of the largest coastal lagoons in RI. The study site lies immediately west of the Charlestown Breachway (constructed in the 1950s to permanently connect the pond with the Atlantic Ocean) and is entirely within the South Shore Management Area owned by the RI Department of Environmental Management.

The study site represents approximately 15% of the estuarine wetland within Ninigret Pond. It includes low and high salt marsh and coastal upland habitats (Fuss & O’Neill, 2015). Low marsh is dominated by tall-form *Spartina alterniflora*, and high marsh consists of *Spartina patens*, *Distichlis spicata*, and *Juncus gerardii*, with *Iva frutescens* and invasive *Phragmites australis* found at higher elevations near the upland. An 8.9-ha subsection of the overall study site exhibits signs of SLR and inundation stress including large areas of marsh platform die-off, in both low and high marsh. Die-off areas are distinct from natural pools and pannes in that they are shallow impounded surface depressions containing combinations of bare peat, open water, stunted vegetation, and filamentous algae (Kutcher et al., 2022). In other areas, stunted *S. alterniflora* intermixes with high marsh grasses, providing evidence of ongoing SLR effects on the marsh. Because of these conditions and concern over high marsh loss in the region (Raposa et al., 2017a), this subsection of the site was the target of sediment placement in this project.

The control site is a 3-ha back-barrier salt marsh located ~0.6 km west of the study site on the southern edge of Ninigret Pond. Similar to initial conditions at the study site, it includes a mix of tall- and short-form *S. alterniflora* and high marsh grasses (*S. patens* and *D. spicata*), and is bordered at the upland edge by *I. frutescens*/*J. gerardii* and *P. australis*. The marsh is unditched with a small number of remnant pools on the marsh platform. The control marsh is also exhibiting SLR stress including stunted vegetation, waterlogged soils and expanding marsh surface die-off areas (Kutcher et al., 2022). Surface salinity can exceed 35 ppt, likely due to tidal water evaporation in shallow die-off areas (Mikula et al., 2019).

### Restoration activities

#### Restoration planning and implementation

The project concept was developed by the RI Coastal Resources Management Council in partnership with the Town of Charlestown, Save The Bay, Salt Ponds Coalition, and the U.S. Fish and Wildlife Service. Project design was driven by restoration goals and construction considerations, with key drivers being the estimated volume of material needed to achieve target elevations to promote desired marsh plant species and build elevation capital, and the volume of material contained within the areas to be dredged. The study site was segmented into marsh restoration “units” for design purposes, which were designed to have higher elevations at their centers (based on target elevations derived from observational data, *see* below), with gradual slopes extending out to existing marsh elevations. This was to facilitate drainage and avoid shallow-water ponding on the marsh surface at low tide.

To determine growth elevation ranges of dominant marsh plant species and develop elevation targets and ranges for marsh restoration unit design, high-resolution elevation surveys of the study site were conducted using RTK in November 2014 and April 2015, with key plant species and habitat types recorded at each survey point. Maximum target elevations were then established by projecting the upper end of the growth ranges of target species under an intermediate to low SLR scenario of 11.27 cm by 2040 (Fuss & O’Neill, 2015). The resultant target elevation range for high marsh species (*S. patens*, *J. gerardii*, and *D. spicata*) was 17.78–25.40 cm NAVD88, and 25.44–30.48 cm NAVD 88 for *I. Frutescens* (a range was not determined for *S. alterniflora*; it was assumed this species would recover at lower elevations as the new sediment sloped towards existing marsh). Maximum target elevations were then increased by 2.54 cm to account for anticipated compaction of existing sediment under placed sediment based on bulk density measurements of existing marsh soil cores (Fuss & O’Neill, 2015). Added sediment thickness was targeted to range from 10 to 30 cm, but soil samples from 2019 show that depths ultimately ranged from 10 to 48 cm, with thicker depths found on former die-off areas that compacted under added sand, thus requiring more new sediment to reach elevation targets. Seven additional soil core samples taken from the dredging area prior to this project revealed that the substrate was primarily composed of sand particles (<12% silt/clay). Grain-size data from a previous sedimentation-rate study also revealed that the areas to be dredged were composed of sand-sized particles (Fuss & O’Neill, 2015).

Dredging and sediment placement occurred between November 1, 2016 and January 31, 2017 to avoid impacts to fish and wildlife. Sediment was removed from the channel by two hydraulic dredges and discharged *via* 20-cm pipe onto the existing marsh surface. Placed sediment was then graded by low ground-pressure bulldozers to target elevations that were marked on stakes throughout the project site. At the beginning of the first growing season, marsh-plant plugs (primarily *S. alterniflora* and *D. spicata*, with lesser amounts of *Baccharis halimifolia*, *I. frutescens*, *J. gerardii*, *Panicum virgatum*, *Solidago sempervirens*, and *S. patens*) were planted in low marsh, high marsh, and higher-elevation habitats to facilitate revegetation. Combining all species, approximately 80 planted areas were established across the marsh in 2017, totaling ~1.4 ha (Figure 1); within each area, plugs were planted 30–45 cm apart on center.

#### Adaptive management of marsh hydrology and platform micro-topography

After completion of dredging and sediment placement project phases, the first period of adaptive management commenced. In February 2017, bare-root culms of American beachgrass (*Ammophila breviligulata*) were planted at higher elevations, and fencing installed, to trap wind-blown sediments and limit sediment loss into existing channels. Prior to the first growing season, ~235 m of creeks (45-cm deep and 60-cm wide) were excavated with a low ground-pressure excavator to restore tidal hydrology in the sediment placement area, with significant grading required to stabilize creekbank slopes. Runnels (small, shallow channels generally less than 30-cm wide and deep; Besterman et al., 2022) were also dug by hand during this time to facilitate drainage of fresh and brackish water from high-elevation depressions in the sediment placement area, and to tie into upland areas where groundwater was trapped by the new sediment. Adaptive management of hydrology was guided in part by marsh-scale salinity mapping that identified high salinity areas and locations of freshwater inputs (Supplementary Figure S1). All initial adaptive management activities were completed by early June 2017 to avoid impacts to nesting birds.

Another period of adaptive management began in July 2017 and continued for approximately 16 months through 2018. Activities during this phase included *P. australis* management, additional grading, maintenance of existing creeks, creation of new runnels, and additional plantings. *Phragmites australis* that was invading the new sediment placement area was hand-pulled during the first and second growing seasons, and established stands adjacent to the sediment placement area were treated with herbicides (imazapyr and glyphosate) in September 2017 and then spot treated in fall 2018. Additional areas were targeted for herbicide treatment in 2018 where *P. australis* established after sediment placement. Existing creeks were maintained by removing sand that filled the creeks from lateral erosion or from the new marsh surface *via* wind, and by regrading and planting creek banks with *S. alterniflora* to help stabilization. To drain groundwater and impounded freshwater along the upland border of the marsh and discourage *P. australis* colonization, additional elevation adjustments were made *via* excavator-grading and new runnels were excavated. Runnels were also dug in lower elevation areas to drain salt water impounded by the marsh-peat sill along the edge of the sediment placement area. All told, 235 m of new creeks and 1760 m of new runnels were excavated or hand dug after sediment placement at the impact site. An additional 0.15 ha of plugs were planted in 2018.

After approximately three growing seasons, as vegetation recolonization increased and hydrology began to stabilize, adaptive management became less frequent. Beginning in 2019, such activities were generally limited to hand maintenance of existing creeks and runnels and additional *P. australis* pulling.

### Monitoring

Our general approach was to monitor ecological parameters in the study marsh before and after sediment placement to quantify change over time and use similar monitoring data from the control marsh to account for natural levels of interannual change to isolate changes in the study marsh due to sediment placement (i.e., a before-after-control-impact [BACI] design; Stewart-Oaten et al., 1992; Underwood, 1992; Stewart-Oaten, 2003). Using this design, the sediment placement marsh is hereafter referred to as the impact site. Due to personnel limitations, monitoring duration and frequency varied among parameters (e.g., some were not measured in the control marsh, others were not measured every year), but our overall annual monitoring window generally spanned 5–6 years. Two years of data were collected before sediment placement (2015, 2016), and most parameters were monitored for an additional 3 years (2017–2019) to quantify immediate and short-term change, although a few key parameters were also monitored in 2020. We monitored a diverse suite of parameters to evaluate ecosystem responses to sediment placement (Table 1). Specific descriptions of the monitoring timeline and methods for each parameter are described below.

#### Elevation

Elevation surveys of the impact site were conducted in 2014 (before sediment placement) by the Town of Charlestown, and in 2017 (immediately post sediment placement) and 2018 by the University of RI after adaptive management activities. Surveys were conducted on the ground using survey-grade (dual-frequency) GNSS receivers (2014) and a robotic total station (2017 and 2018). The total station surveys were initiated from benchmark locations derived from GNSS observations. Thus, all topographic data have comparable accuracies (centimeter). All topographic data were referenced to the RI State Plane Coordinate System NAD 83 (2011) (FIPS code 3800) with vertical coordinates referenced to the North American Vertical Datum of 1988 (Geoid 12B). The 2014 survey had an average point spacing of 3,030 pts ha^−1^ while the 2017 and 2018 surveys had average point spacings of 244 and 596 pts ha^−1^, respectively. All surveys were conducted in a grid pattern to accurately represent micro-topographic changes at the site. Topographic data from all three surveys were interpolated in ArcGIS (ESRI, 2021) and digital elevation models were created. The surveys were standardized for comparisons with each other by creating equal-resolution DEMs (61-cm pixel size) and clipping the extents of each DEM with a single polygon. One survey of the control site was conducted in winter 2018 using the same methods as at the impact site in 2018. The 2018 surveys of both sites included the collection of a single elevation point from the center of each plot established for vegetation monitoring and, to compare with pre-sediment placement, elevation from the center of each impact marsh plot in 2014 was estimated from interpolation as described above.

In addition to broad-scale mapping, finer-scale elevation changes were monitored at three locations in each site using deep-rod surface elevation tables (SETs; Cahoon et al., 2002a; Cahoon et al., 2002b). In the impact site, locations were randomly selected within 2–3 m of established vegetation monitoring transects, and SETs were monitored annually in early fall, with additional samples collected in spring 2018, 2019, and 2020. SETs were monitored in the control marsh annually from 2015 to 2017 and again in 2020. For each sample, measurements were taken from nine pins in each of four opposite directions (36 pins total). Two 50 cm × 50 cm feldspar clay marker horizons were also established within each impact marsh SET in 2015 and measured in fall 2018 to calculate short-term surface accretion rates.

#### Hydrology

To develop a local tidal datum and calculate mean high water (MHW) applicable to both the impact and control sites, a HOBO U20-001-04 water level logger (Onset Corp.) was deployed from March 30 - September 24, 2018 in the coastal pond adjacent to the impact site and programmed to record water height above the sensor every 6 min. The logger was housed in a PVC tube (which was mounted to a cinder block that was pinned with stainless steel rods driven 1 m into the substrate) and attached to the end of a PVC housing cap. Geodetic height of the sensor was determined by GNSS surveying of a target mounted to the top of the cap, ensuring that measurements were always taken from the same location. Water height above the sensor was also measured during deployment and periodically over the 6-month period to serve as an accuracy check and account for movement and pressure-sensor drift. Tidal datums for the deployment period were calculated by using the Tidal Analysis Datum Calculator (NOAA CO-OPS). Values were converted to NAVD88 using the mean of GNSS measurements on the mount target at deployment and retrieval. These tidal datums represent conditions specific to the deployment period and have not been tied into a long-term control station.

To examine interannual patterns in marsh surface inundation and drainage, water heights were monitored annually at two random locations in each marsh for approximately 1 month each summer using HOBO loggers (same model as above) deployed in PVC wells. Each 0.75-cm long × 20-cm diameter well was perforated with 6-mm holes for drainage and sunk into the marsh until 10 cm remained above the marsh surface. Loggers were suspended to a fixed depth near the bottom of each well and programmed to collect overall pressure data (converted to water depth by compensating for ambient barometric pressure) every 15 min. At the beginning of each deployment, distances (cm) from *1*) the uncapped well top to the marsh surface, and *2*) the well top to the sensor were measured and used to calculate marsh surface inundation (% of time tidal water was on top of the marsh) and drainage (percent of time water was lower than 5 cm below the marsh surface).

#### Soil characteristics

In August 2015 (pre-sediment placement) and 2019 (post-sediment placement), twenty permanent plots (10 in each site) were sampled to assess soil % moisture, bulk density, % organic matter, and belowground biomass (i.e., roots and rhizomes). Plots were selected along the existing vegetation monitoring transects to be representative of the marsh landscape including low and high marsh zones. A McCauley peat sampler was used to collect samples to a depth of 25 cm at the control and impact sites in 2015, and the peat sampler was again used in 2019 at the same plots at the control site. A combination of soil pits (to sample the added sediment) and peat sampler (for collecting from the underlying peat) were used at the impact site in 2019. Core and soil samples were stored in a freezer until processing and laboratory analyses.

Defrosted soil cores were cut in 5-cm increments, and wet and dry weights were used to calculate % moisture and bulk density, respectively. To determine organic matter and carbonate content, unsieved sediment samples were dried, ground with a mortar and pestle, and subsamples were weighed into crucibles (~5 mg) for loss on ignition (Heiri et al., 2001). Dried samples were then heated to 550°C for 4 h, cooled, and reweighed. Percent organic matter (% OM) was calculated as 100*(1−(weight after 550°C/initial weight)). Because of noticeable shell content in the dredged material, % carbonate was measured at the impact and control sites in 2019. After determining % OM, subsamples were then heated to 950°C for 2 h, cooled and reweighed to determine carbonate content, where % carbonate was calculated as 100*(1−(weight after 950°C/initial weight)). Roots and rhizomes representative of the top 25 cm of soil were estimated by sieving a soil subsample through a 1-mm sieve.

#### Soil shear strength

Soil shear strength was measured vertically with depth directly adjacent to vegetation monitoring plots within 2 h of low tide to allow for comparisons of marsh soil strength at similar tide heights between sites. Marsh soil strength reflects the resistance to shearing stresses afforded by the cohesion and frictional resistance of soil constituents and is presumed to be an indicator of soil matrix integrity in salt marshes (Turner et al., 2009; Howes et al., 2010; Turner, 2011). A field-vane shear tester (AMS part 59020, American Falls, ID, United States) was used to measure the minimum shear strength in kilopascals (kPa) required to force soil failure (Swarzenski et al., 2008; Turner et al., 2009; Graham and Mendelssohn, 2014). Shear strength was measured at six depths, beginning at 10 cm, and in 10-cm increments thereafter. In some plots, fewer measurements were taken because the field-vane could not penetrate the sandy or mineral soil at depth. Shear strength values obtained with a field vane may overestimate marsh soil strength because of strain rate, anisotropy within the soil, and rod friction, but field-vane measures of salt marsh shear strength have been used for comparative purposes (e.g., Turner et al., 2009, 2017; Howes et al., 2010; Graham and Mendelssohn, 2014).

#### Vegetation cover

Five or six monitoring transects were established at each marsh. Transects spanned the entire marsh platform from open water to the marsh/upland ecotone. Vegetation was monitored in 1 m^2^ plots located equidistant along the transects (21 plots at the impact site; 20 at the control) at the height of the growing season in 2015 and 2016 before sediment placement and annually thereafter through 2020. None of the plots at the impact marsh were in areas that were planted with vegetation as part of adaptive project management. On each date, cover of vegetation species and other features (bare ground, open water, wrack) was quantified in each plot in both marshes using the point intercept method with 50 points per plot (Roman et al., 2001) and converted to % cover. In all plots each year, stem densities were non-destructively estimated with a 0.25 m^2^ sub-quadrat within the main plot for *S. alterniflora* and *P. australis*, and with a 0.1 m^2^ sub-quadrat for *S. patens*, *J. gerardii*, and *D. spicata*. Also, within each main plot, 16 shoots of each of these five species (when present) were randomly measured for length. Stem density and mean length measures were used to estimate aboveground biomass using stem length–biomass regression analyses generated from samples harvested from the study sites in 2015 (next section).

#### Stem length–biomass relationships

To determine the relationship of stem length to biomass for each of the five dominant species, 0.25 m^2^ quadrats for *S. alterniflora* and *P. australis* and 0.1 m^2^ quadrats for *S. patens*, *J. gerardii*, and *D. spicata* were used to harvest representative samples of each species at the impact and control sites in 2015. A total of 25 quadrats (five per species) were collected at each site. Individual stem length and dry weight were measured and used in regression analyses to determine a specific stem length-biomass relationship for each species (Supplementary Table S1).

#### Nekton

Nekton communities (fishes and decapod crustaceans) were sampled in the impact and control sites using 1 m^2^ throw traps following the methods in Raposa et al. (2003). Sampling was conducted annually in July and September to account for seasonal community shifts. Each sampling period, 20 samples were collected in each marsh from permanent locations that were randomly selected from all shallow aquatic habitats (creeks, pools, impounded die-off, and marsh edge). After sediment placement, however, only 13 stations were sampled in the impact marsh each period; seven others (mostly former die-off areas) were filled by sand. Sampling was always conducted when tidal water was drained off the marsh surface. All nekton captured were identified to species, counted, and released. Crab burrow density was also determined by counting all crab burrows found within a 0.625-m^2^ quadrat placed on the marsh surface within 0.5 m of the seaward edge, next to each throw trap sample.

#### Birds

Four bird survey points in the impact site were selected using a generalized random-tessellation stratified (GRTS) sampling system in package “spsurvey,” program R (Kincaid et al., 2019; R Core Team, 2019). Eight survey points in the control marsh were randomly selected from existing survey points that were established for the Saltmarsh Habitat & Avian Research Program (SHARP), the U.S. Fish and Wildlife Service Salt Marsh Integrity program or a historic marsh bird survey (Shriver et al., 2004; Neckles et al., 2013; Shriver et al., 2015; Wiest et al., 2016). All impact and control points were surveyed 2 years before (2015, 2016) and 2 years after (2018, 2019) sediment placement, with two to three surveys conducted at each point from early May to mid July each year.

Field surveys followed the North American Marsh Bird Monitoring Protocol (Conway, 2011) to collect bird count data (an indicator of abundance) and estimate bird densities at both sites. A single observer conducted surveys within the period extending from 30 min before sunrise to no later than 11: 00 AM. At each point on each date, a 5-min passive point count was conducted, divided into 1-min intervals, during which the number of individuals of each species detected within 100 m of the survey point was recorded. Surveys were not conducted during high winds, or heavy fog or rain.

### Data analysis

#### Elevation change

A Paired *t*-test was used to compare monitoring plot elevations in the impact marsh before (interpolated 2014 data) and after (2018 RTK data) sediment addition, and Kruskal-Wallis one-way ANOVA on ranks was used to compare these two sets of data with the control marsh in 2018. To explore patterns in marsh elevation change rates from SETs before and after sediment placement, we calculated mean pin height from each SET on each sampling date in both marshes. Means were first calculated separately for each of the four SET arm directions, and then averaged across arms.

#### Soils

A three-factor ANOVA model (year, site, and depth factors) was used to examine differences in moisture fraction, bulk density, and % OM. When the three-factor interaction term was significant at alpha 0.05, pairwise comparisons between sites were year and depth-specific, and pairwise comparisons between years were site and depth-specific, with Bonferroni adjustment made due to the five depth-specific comparisons. Two-factor models were used for the analysis of belowground biomass (year and site factors) and 2019 carbonate fraction (site and depth), using log-transformed data. Prior to carbonate data analysis, half the minimum value observed was added to each value to eliminate zero valued results.

To analyze soil shear strength, two depth increments (averaged across 10-cm intervals) were examined: 10–30 cm and 40–60 cm. The 10–30 cm interval is the usual depth associated with root and rhizome activity and the 40–60 cm zone is the sub-rooting depth (Wigand et al., 2018). Only depths where shear strength measures were obtained were used in the analyses. To meet statistical assumptions, shear strength measurements were log-transformed (adding 1 to all values first), and transect means were calculated for the given year and depth prior to analyses. Statistical analyses were performed using a two-factor ANOVA, with site (control, impact) and time as factors. The time factor was defined by years before (2015–16) and years after sediment placement (2017–20). Separate models were run for the two depth categories (10–30 cm and 40–60 cm).

#### Vegetation

For most analyses, bare and open water cover were summed into an aggregate “unvegetated cover” category because we were interested in total revegetation responses. Although, for cover ANOVA analyses only, bare and open water were also examined individually to explore specific aspects of recovery.

Cover analyses were performed for total plant cover, dominant two species per plot, high marsh dominants (*S. patens*, *D. spicata*, *J. gerardii*), successional colonists (*Spergularia marina*, *Suaeda* spp., *Atriplex* spp., and *Salicornia spp.*), selected individual plant species, bare ground, open water, and total unvegetated using transect mean percent cover values in a two-factor ANOVA with time and site factors. The model included an evaluation of the site × time interaction term, consistent with the BACI design. The time factor was broken out with pre-sediment placement as years 2015 and 2016, and each post-sediment placement year evaluated separately. This differential treatment of years allowed us to examine vegetation trajectory over time after sediment placement, and pre-sediment placement years at both sites allowed for examination of natural, interannual variability. Evaluating the significance of the site × time interaction allowed us to assess whether a significant change over time occurred at one site, but not the other, as well as whether the impact site differed from the control site each year after sediment placement. When the interaction was significant, time comparisons were done by comparing the site-specific pre-sediment placement category separately with each post-sediment placement year for that site, but not directly comparing post-sediment placement years to each other, using Bonferroni correction among site/time combinations. When the interaction was not statistically significant, the main effects of time and site were examined.

Stem density and aboveground biomass of five dominant plant species (*S. alterniflora*, *S. patens*, *D. spicata*, *J. gerardii*, and *P. australis*) were examined to assess change over time at each site, before and after sediment placement. Statistical analyses generally followed the same model approach and time categorization used for vegetation cover analyses. As with cover data, stem density and biomass were analyzed based on comparisons using transect means; however transect means were calculated using natural log(x+1) transformed count/biomass values. Since *P. australis* was so infrequently detected on vegetation transects at the impact site, a presence/absence evaluation was used in addition to transect mean analyses, using a logistic regression model. For this comparison, separate models were fit using indicator variables (i.e., binary) for each post-sediment placement year, and for interaction terms between year and site. Models were then fit with and without the time/site interaction terms, and with and without the time variables, and used likelihood ratio Chi-Square tests comparing models to evaluate overall significance of the interaction and of the time factor.

For all cover ANOVA analyses, transect means rather than plot measurements were used due to the high frequency of zero-valued results and other ties at the plot level having a strong impact on the constant error variance and other statistical assumptions required for the analyses. Statistical significance was assessed for all models at the overall 95% confidence level.

A two-factor crossed PERMANOVA was used to test for differences in vegetation community composition between sites and over time using resemblance matrices constructed with Bray-Curtis similarity between samples, and square-root transformed data to dampen the effects of highly-abundant species. A significant time × site interaction term was used to identify significant changes that occurred at the impact site and not the control site as indicators of sediment placement effects. Significant changes at the impact site were followed by pairwise comparisons among each pair of years, followed by similarity percentages (SIMPER) to identify species or cover classes that contributed most to overall community similarity each year in each marsh. Finally, metric multidimensional scaling (MDS) was used to visualize spatial patterns of vegetation communities within and among years in both marshes using bootstrap average analysis on Bray-Curtis similarity matrices; group averages were calculated from *n* = 60 bootstrap averages for each year and overlain by shaded 95% confidence regions (Clarke and Gorley, 2015).

#### Fauna (nekton and birds)

The same multivariate analyses (i.e., two-factor PERMANOVA, SIMPER, *etc*.) described above for vegetation were also used to test for changes in nekton and bird communities over time and between sites, using the interaction term to identify changes at the impact site related to sediment placement. The only difference is that nekton data were fourth-root transformed prior to analysis to dampen effects of extremely abundant species (i.e., daggerblade grass shrimp *Palaemonetes pugio*); bird count data were square-root transformed.

Nekton and crab burrow densities typically had high frequencies of zeros and were therefore analyzed using logistic regression models with significance of the two factors and their interaction evaluated using Likelihood Ratio Chi-Square tests. However, total nekton, sheepshead minnow (*Cyprinodon variegatus)*, and mummichog (*Fundulus heteroclitus*) densities, which had fewer zeros, were analyzed using two-factor ANOVA and Normal score transformation (i.e., ranking each result and transforming the relative ranks to quantiles of the standard normal distribution). A time-series plot was also created to visualize changes in total nekton density and total nekton population size (calculated as mean density × total area of creek, pond and pool habitat) over time at both sites. For each species/station/year, mean density from the July and September sampling periods was used for all nekton analyses.

Log(x+1) transformed bird count data were analyzed using two-factor ANOVA (site and time) with pairwise time comparisons conducted using Bonferroni correction. Bird species and guild densities in the impact and control sites were also estimated for each survey year from the raw count data using the package “unmarked” in R using a generalized multinomial N-mixture model (the “gmultmix” function) (Royle, 2004; Fiske and Chandler, 2011; R Core Team, 2019). Each species’ detection probability (p) was accounted for with the time-of-detection method using the five 1-min intervals (Farnsworth et al., 2002). As with nekton, time-series plots were then created to visualize changes in the densities of all birds combined and of major guilds over time. Unless otherwise specified, all statistical analyses were performed using SAS Version 9.4 software (SAS Institute Inc, 2013).

## Results

**FIGURE 2:**
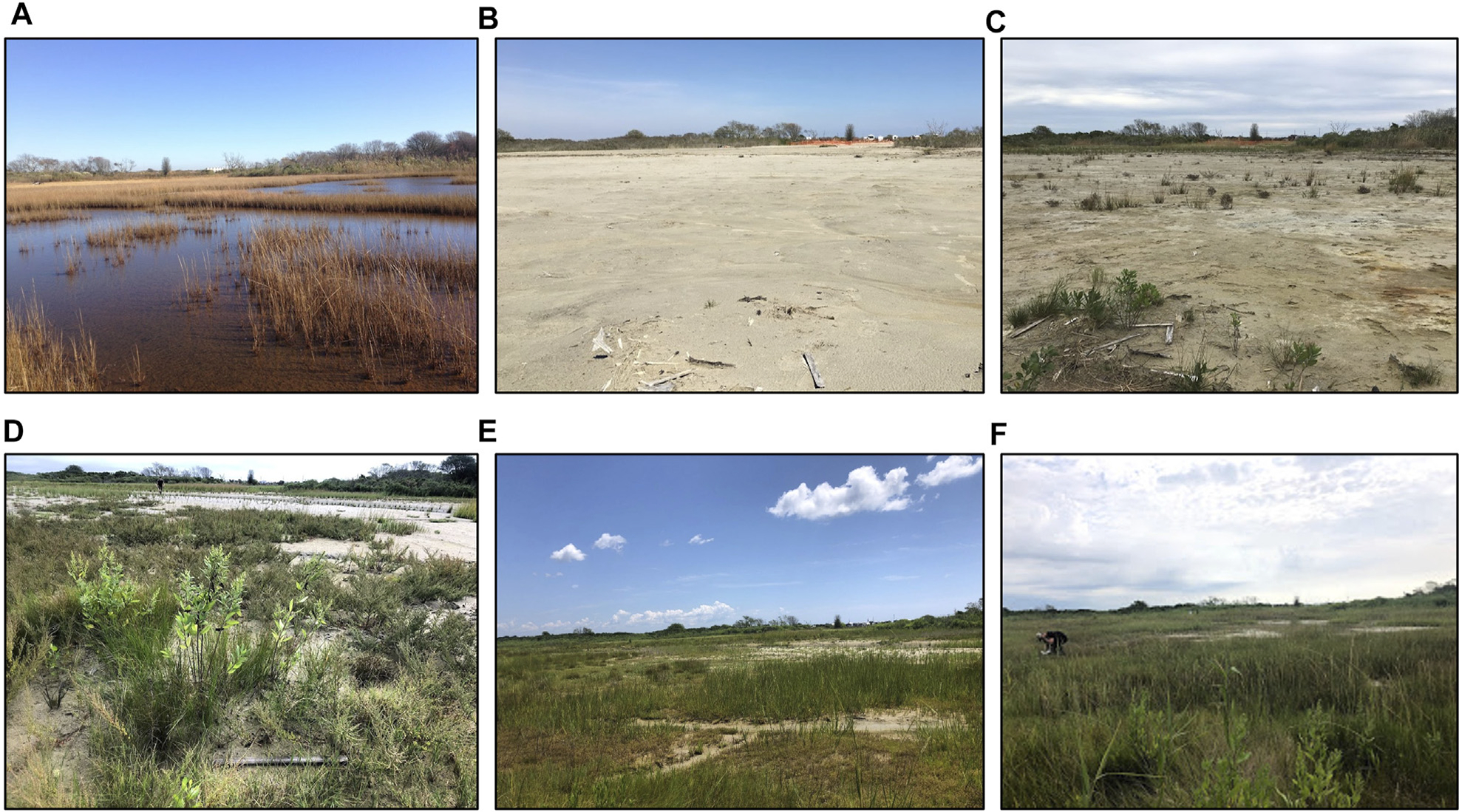
Time series photographs of the Ninigret sediment placement marsh. Initial conditions **(A)**, immediately after sediment placement **(B)**, first growing season after sediment placement **(C)**, and second **(D)**, third **(E)** and fourth **(F)** growing seasons (2018–2020).

The placement of sediment on the Ninigret impact site during winter 2016 altered the landscape from a waterlogged marsh with stressed vegetation and expansive surface ponding to one dominated by sand, similar to overwash of a barrier beach, with most waterlogged and die-off ponded areas filled in with new dredged sand (Figure 1). Marsh vegetation recovered rapidly to initial levels after sediment placement *via* natural colonization and plantings. By 2019, the third growing season after sediment placement, much of the impact site had re-vegetated (albeit sparsely) with a mix of saltmarsh, pioneer, brackish, and upland salt-tolerant species, and by 2020 the majority of the site was vegetated, with very little exposed sand (Figure 2).

### Elevation

**FIGURE 3:**
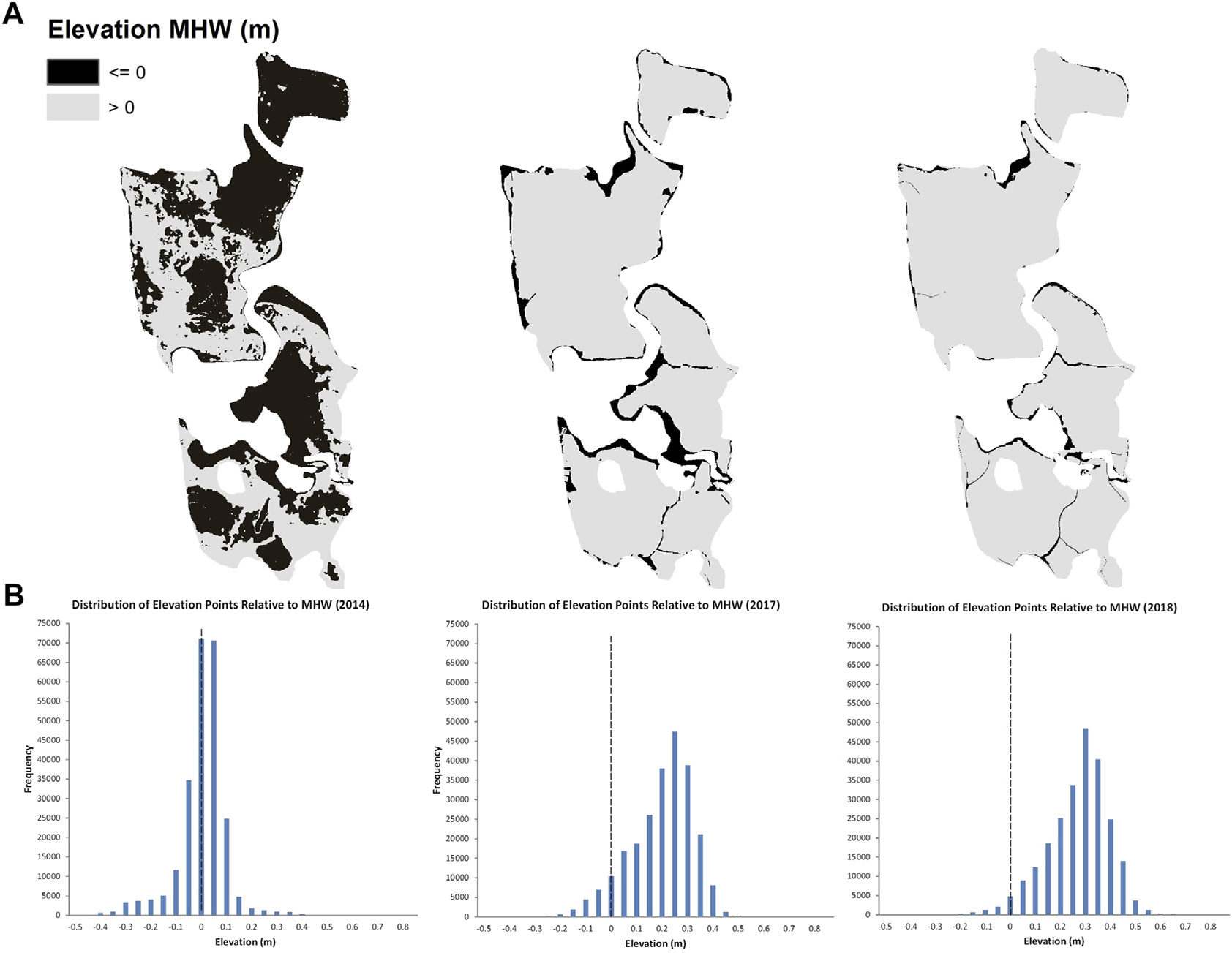
Whole-marsh scale changes in elevation **(A)** and frequency distributions of elevation points from field surveys **(B)** at the Ninigret impact site over time. Left to right: 2014 = pre-sediment placement conditions when over half the marsh area was at elevations below MHW; 2017 = the first growing season after sediment placement when elevation was greatly increased by new sand; 2018 = the second growing season after sediment placement when elevation above MHW again increased slightly.

Sediment placement was effective for raising the elevation of the impact marsh surface and increasing elevation capital relative to water levels, with enhanced elevations appearing stable after 3 years. From the survey before sediment placement, only 44% (39,284 m^2^) of the marsh was at elevations above MHW (calculated as −0.112 m NAVD88; Figure 3). By 2017, the first growing season after sediment placement, 88% (80,585 m^2^) of the marsh was above MHW; by 2018 the site gained slightly more elevation capital, with 96% of the marsh (86,256 m^2^) above MHW. Surface elevations in vegetation monitoring plots in 2018 averaged 0.22 m MHW (range of −0.08 to 0.35 m MHW) at the impact site and 0.03 m MHW (range of −0.16 to 0.13 m MHW) at the control site. Plot elevations at the impact site before sediment placement in 2014 averaged 0.003 m MHW (range of −0.15 to 0.18 m MHW). Plot elevations were significantly higher after sediment placement at the impact site compared to initial conditions (*t*-test, one-tailed *p* < 0.001) and when comparing all three sets of plot elevation data, control site elevations in 2018 were not significantly different than the impact site before sediment placement, but were significantly lower than the impact site after sediment placement (ANOVA, *p* < 0.001).

**FIGURE 4:**
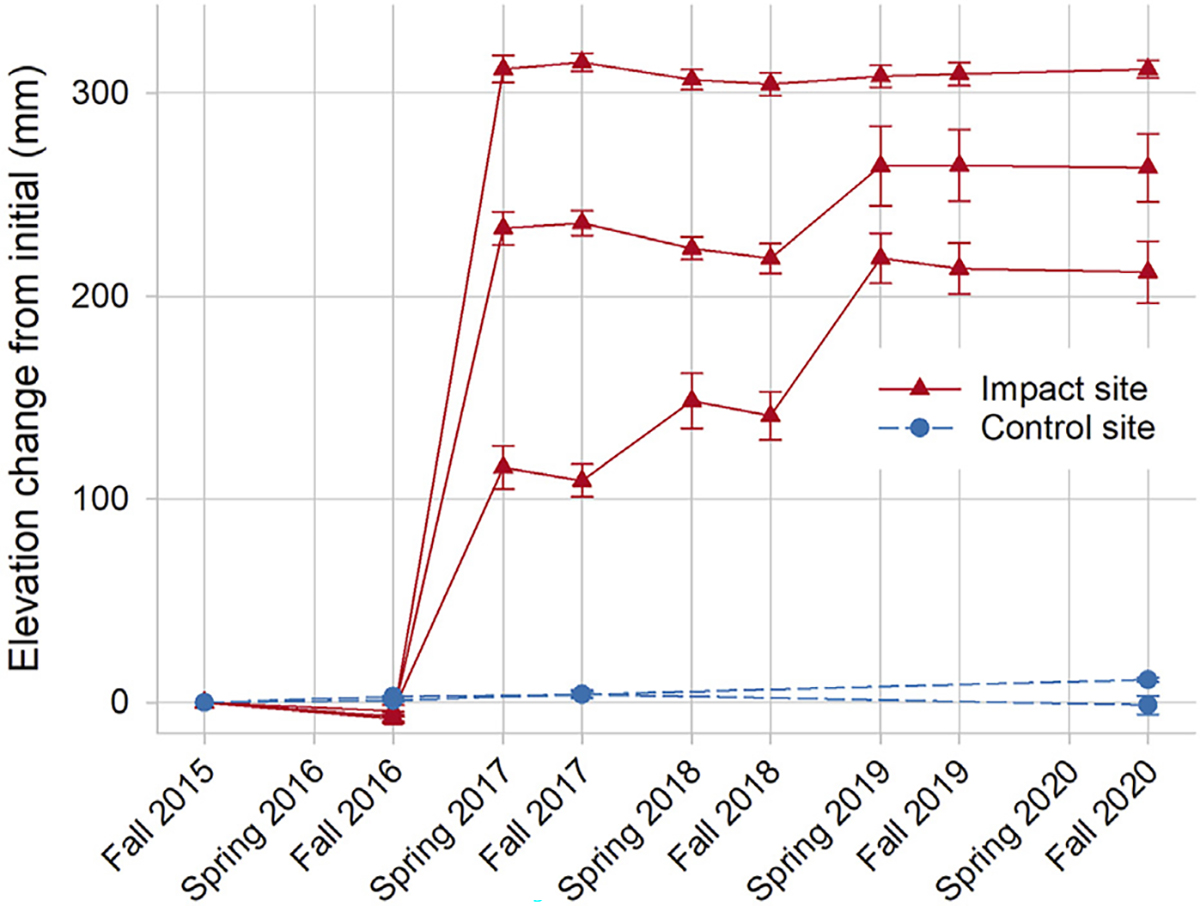
Change in marsh elevation over time relative to initial conditions (fall 2015) from three SETs each in the impact and control marshes. Sediment placement occurred in winter after the fall 2016 measurements. Error bars are 1 SE.

Finer-scale changes in elevation were discernible from SET monitoring, which also demonstrated a large increase in elevation capital immediately after sediment placement, and enhanced rates of annual elevation gain thereafter. Impact marsh elevation increased by a mean of 227 mm (range = 120–320 mm) across the three SETs from 2016 to 2017 (Figure 4). In the 4 years following sediment placement, the mean rate of elevation gain was 20.76 mm yr^−1^, which was noticeably higher than the mean rate prior to sediment placement (−6.40 mm yr^−1^) and the overall rate in the nearby control marsh (1.29 mm yr^−1^; 2015–2020). This high rate, however, was likely due to unconsolidated sand movement on the new marsh because the mean rate of elevation gain in the impact marsh from spring 2019 through fall 2020, when the marsh revegetated and appeared to stabilize, was only −0.84 mm yr^−1^.

The overall increase in elevation after sediment placement was lower than optimal due to below-ground subsidence. Marker horizon readings in fall 2018 showed that a mean of 284 mm of sediment (range = 187–344 mm) was added to the impact marsh as of October 2018. From SET readings, the mean net elevation increase during this same time period was 221 mm (range = 141–304 mm), indicating that mean below-ground subsidence was 63 mm (range = 40–103 mm). Subsidence was apparently due to sediment placement on existing degraded peat. The largest post-sediment placement elevation increases were measured in spring (2018 and 2019) at the SET nearest the open pond. This was also the only SET with rapid vegetation regrowth, suggesting that vegetation trapped unconsolidated sediments from the project that were moving *via* winter northerly winds.

### Hydrology

**FIGURE 5:**
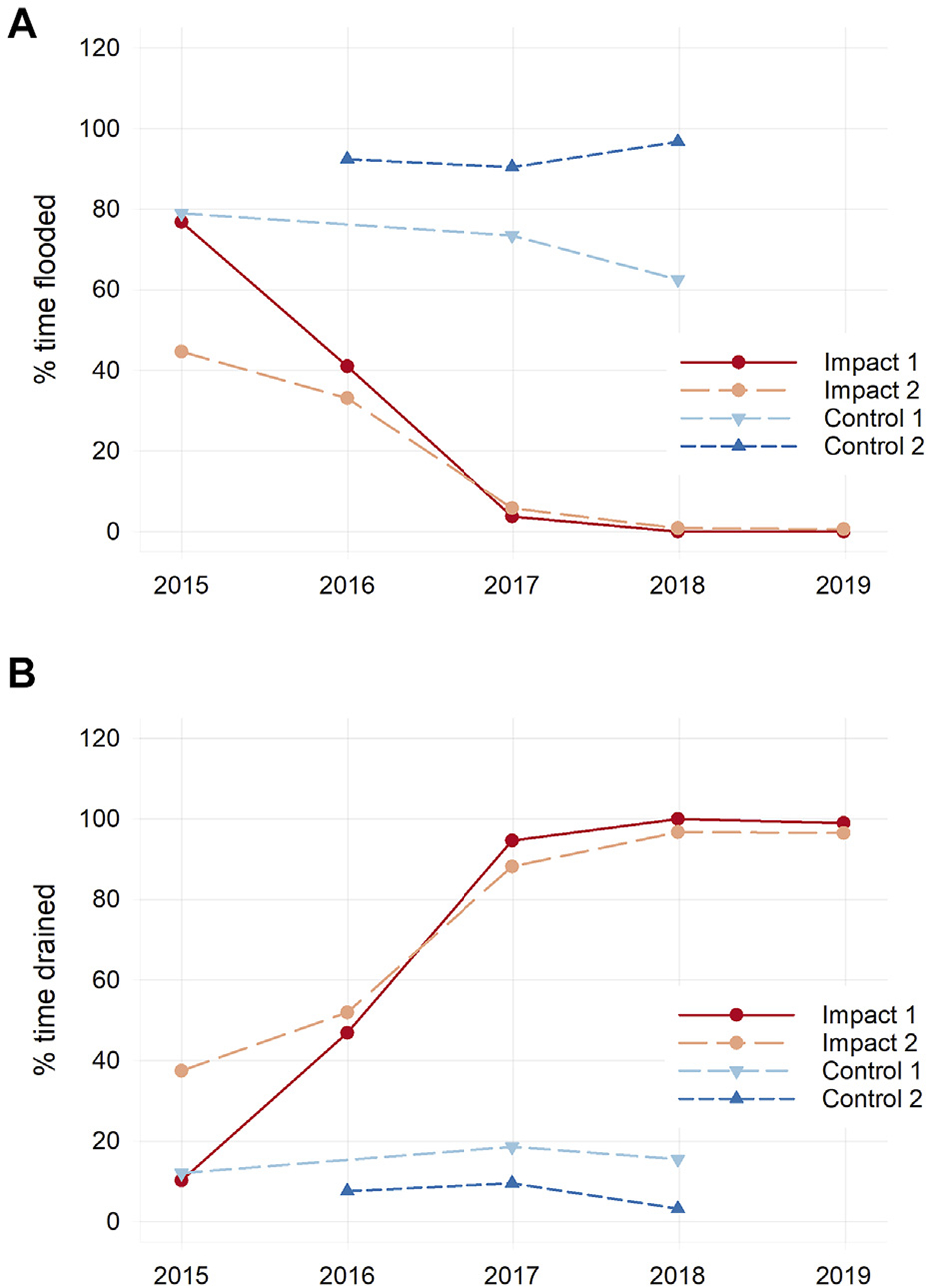
Percent of time each year that the marsh surface was inundated with tidal water **(A)** and drained (i.e., water levels less than 5 cm below the surface of the marsh; **(B)** in the impact and control marshes. Two loggers were deployed simultaneously in each marsh for approximately 1 month in summer each year. Sediment placement occurred between 2016 and 2017.

Sediment placement altered hydrology of the marsh surface and uppermost sediment layers, eliciting a shift from prolonged inundation and persistent soil waterlogging before sediment placement to greatly-reduced inundation and enhanced drainage after. Data from HOBO loggers deployed in wells within marsh soils show that the percent of time the impact marsh surface was inundated with tidal water decreased from 49% before sediment placement (mean from two stations, 2015–2016) to 2% after sediment placement (Figure 5). Belowground drainage was also enhanced; before sediment placement, water levels dropped lower than 5 cm below the marsh surface only 37% of the time on average; this increased to 96% after sediment placement. Surface inundation and belowground drainage patterns were unchanged over time in the control marsh.

### Soils

#### Soil characteristics

Sediment placement altered impact site sediments, which became drier with higher bulk density and a lower fraction of organic matter and root biomass. There was a significant three-factor (year, site, depth) interaction for percent soil moisture (*p* = 0.0221) (Supplementary Table S2). At the control site, there was no difference in soil moisture at any depths between 2015 (before sediment placement) and 2019 (after), while at the impact site, pre-placement was significantly greater than post-placement for all depths. At the control site, mean moisture fraction in 2015 and 2019 ranged from 78 to 79% at 5 cm depth to 62–64% at 25 cm. At the impact site, mean moisture fraction in 2015 ranged from 76% at 5 cm to 53% at 25 cm; in contrast, mean moisture fraction in 2019 ranged from 13% at 5 cm to 30% at 25 cm. In 2019, soil moisture was significantly greater at the control site than the impact site at all depths. The three-factor interaction term was also significant for bulk density (*p* = 0.0252). Impact site bulk density in 2019 (mean ranging from 1.19 g ml^−1^ at 5 cm to 0.93 g ml^−1^ at 25 cm) was significantly greater than at the control (mean ranging from 0.15 g ml^−1^ at 5 cm to 0.56 g ml^−1^ at 25 cm) for all depths except 25 cm. At the impact site, bulk density in 2019 was significantly greater than 2015 (mean ranging from 0.28 g ml^−1^ at 5 cm depth to 0.67 g ml^−1^ at 25 cm) at 5 and 10 cm only, while at the control there was no difference in bulk density between 2015 and 2019 for any depth.

There was a significant two-factor site × time interaction term (*p* = 0.0022) for soil organic matter fraction. At the control site, there was no difference in organic matter fraction between 2015 (0.34 ± 0.06) and 2019 (0.32 ± 0.04), but at the impact site, organic matter fraction in 2015 (0.21 ± 0.06) was significantly greater than 2019 (0.05 ± 0.01). In 2019, control site organic matter fraction was significantly greater than the impact site. There was also a significant two-factor site × year interaction (*p* = 0.0032) for root biomass (to a depth of 25 cm). While there was no difference between 2015 (8008 ± 1409.5 g m^−2^) and 2019 (3266 ± 682.8 g m^−2^) at the control site, root biomass was significantly greater in 2015 (4475 ± 765.9 g m^−2^) than 2019 (461 ± 149.5 g m^−2^) at the impact site. After sediment placement, in 2019, root biomass in the control site was significantly greater than the impact site.

#### Soil shear strength

For the 10–30 cm depth zone, there was a significant site × time interaction (*p* = 0.0042). At the impact site, transect mean shear strength after sediment placement (59.8 ± 5.4 kPa) was 64% greater than before sediment placement (36.6 ± 3.3 kPa). At the control site, there was no difference in mean shear strength between pre-sediment (43.2 ± 4.1 kPa) and post-sediment (37.1 ± 2.9 kPa) placement. Before sediment placement, there was no difference in shear strength between sites, but after sediment placement mean shear strength at the impact site was 61% greater than the control site at 10–30 cm depth. At 30–60 cm depth, there was no difference in shear strength between sites or time periods; transect means ranged from 53.7–68.2 kPa.

#### Vegetation

Both marshes were dominated by perennial graminoids typical of New England salt marshes (*S. alterniflora*, *S. patens*, *D. spicata*, and *J. gerardii*), with *I. frutescens*, *P. australis*, and brackish species in the upper ecotone and unvegetated areas interspersed on the platform (Supplementary Table S3). Few changes were observed in the control marsh, but sediment placement induced dramatic changes in the impact marsh, including ubiquitous bare ground cover the first year after sediment placement, rapid colonization by annual and pioneer species the next 2 years, and recovery of marsh dominants to levels comparable to initial conditions and loss of most bare ground by year four post-sediment placement.

#### Vegetation communities

**FIGURE 6:**
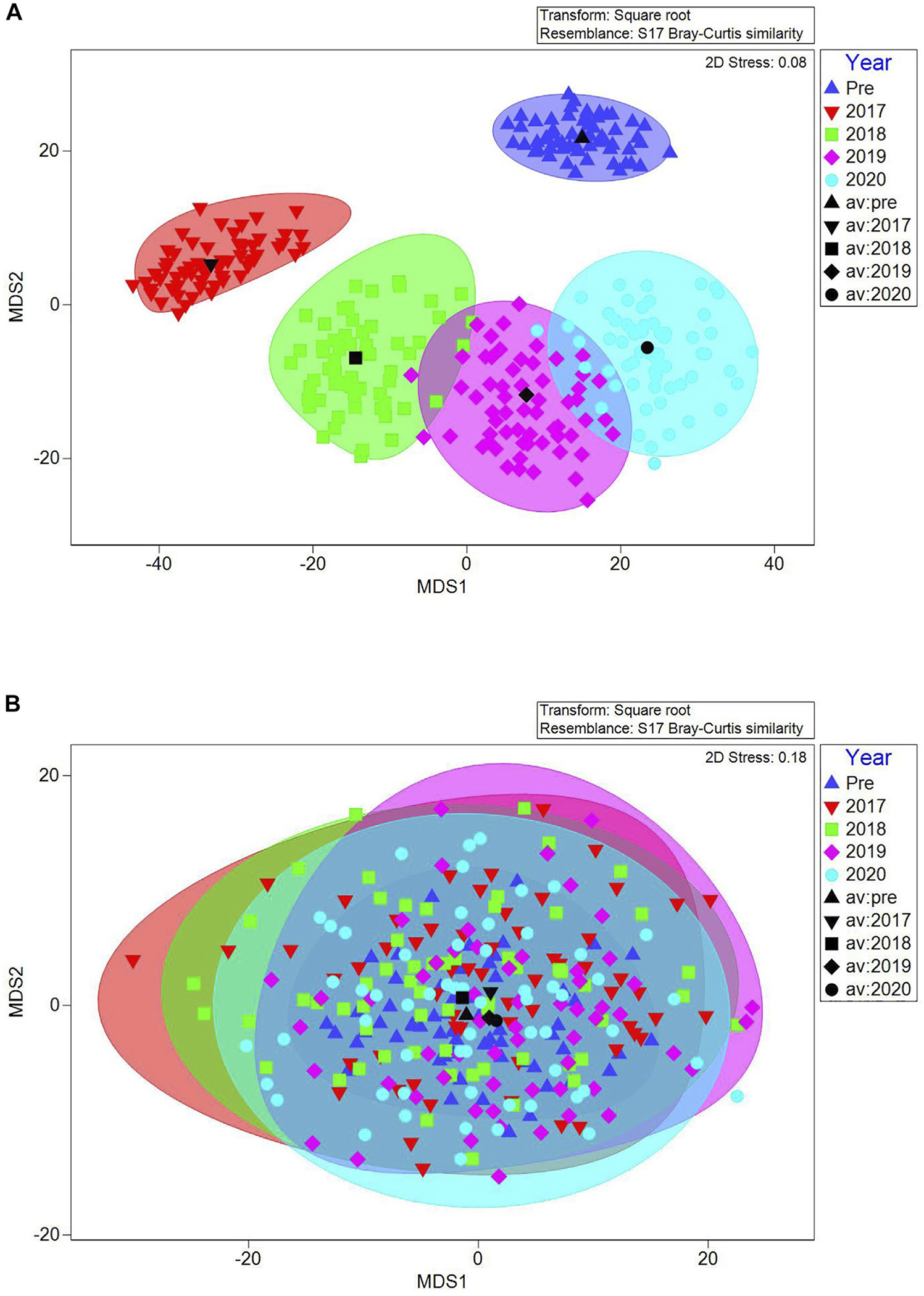
Metric multidimensional scaling of bootstrap averages for vegetation communities in the impact **(A)** and control sites **(B)** over time. Black symbols represent the group average (i.e., the average of the 60 bootstrapped averages) for each year; shaded areas around each group average represent the 95% confidence region estimate for each year.

Vegetation communities were significantly different between sites (PERMANOVA, *pseudo-F* = 13.87; *p* = 0.001) and over time (*pseudo-F* = 3.74, *p* = 0.001). There was also a significant site × time interaction (*pseudo-F* = 3.67, *p* = 0.001) such that vegetation changed significantly over time at the impact site, without concurrent changes at the control site. At the impact site, communities were significantly different between pre-sediment placement years and every post-sediment placement year, and between all pairs of post-sediment placement years except 2019–2020 (Figure 6 and Supplementary Table S4).

Impact site community changes were driven by conspicuous changes in key species as indicated by SIMPER. Before sediment placement, the impact site vegetation community was typified by *S. alterniflora*, unvegetated ground, and *D. spicata*, which contributed a combined 83% to overall similarity. After sediment placement, there were clear changes in plants as the impact site recovered, moving from being overwhelmingly typified by unvegetated ground in 2017, through 2 years of successional changes as marsh plants recolonized, to a 2020 community that was typified by *S. patens* (37%), *J. gerardii* (15%), *S. alterniflora* (11%), and *S. marina* (9%) (Supplementary Table S5). Although never present at the control site and not present at the impact site 2015–2017, pioneer annual plants (*S. marina* and *S. linearis*) colonized bare ground after impact site sediment placement from 2018 to 2020 (Supplementary Table S3); *S. linearis* contributed 12% in 2019 and *S. marina* 9% in 2020 to impact site community similarity. In contrast, communities at the control site were consistently typified by *S. alterniflora*, unvegetated ground, and *D. spicata* every year (these three species contributed to >70% of community similarity each year, except 2018 when *S. patens* also contributed 12%; Supplementary Table S5).

#### Plant cover

**FIGURE 7:**
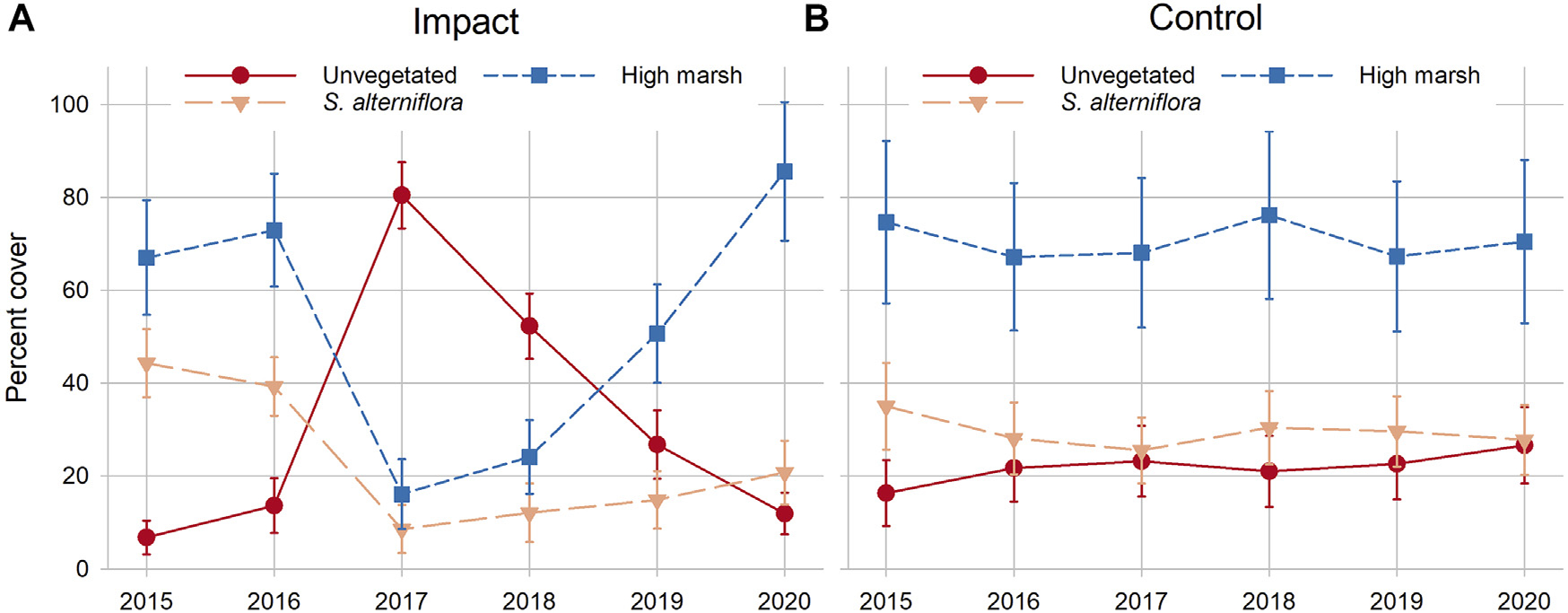
Changes in percent cover of unvegetated ground, Spartina alterniflora and high marsh grasses (Spartina patens, Juncus gerardii and Distichlis spicata combined) over time in the impact **(A)** and control **(B)** marshes. Sediment placement occurred during the winter between the 2016 and 2017 sampling periods. Error bars are 1 SE.

Significant site × time interactions were observed for total vegetation cover, the dominant two species, successional colonists (sum of *S. marina*, *Suaeda* spp., *Atriplex* spp., and *Salicornia* spp.), total unvegetated, and bare ground (Table 2). Total vegetation cover (regardless of species) was significantly lower at the impact site the first year after sediment placement (2017) compared to the impact site pre-sediment placement and the control site. However, there was no difference in total vegetation cover between sites after 2017, indicating rapid colonization and recovery of vegetation by 2018. An inverse pattern was observed at the impact site for bare ground and dominant two species cover—the impact site had significantly more bare ground in 2017 and 2018, the first 2 years after sediment placement, while dominant two species cover was significantly lower these same years (Table 2). By 2019, dominant two species cover at the impact site recovered to pre-sediment placement levels, while the control site stayed largely the same over time. Successional colonist cover at the impact site in 2019 and 2020 was significantly greater than the control site these same years and at the impact site before sediment placement (Table 2). Some individual species showed either overall time or site differences, but no interaction between the two. For example, individual cover of *D. spicata*, *P. australis*, and open water was significantly greater at the control site compared to the impact site throughout the study. Even though there were no statistically significant differences in the cover of any individual high marsh graminoid species between 2020 and pre-sediment placement years, the combined cover of high marsh graminoids (*S. patens*, *D. spicata*, and *J. gerardii*) was trending strongly higher each year after impact site sediment placement, suggesting that cover of these species may not have plateaued 4 years after sediment placement (Figure 7).

#### Stem density and aboveground biomass

The low marsh dominant, tall-form *S. alterniflora*, had a significant site × time interaction, with a significant decrease in stem density (mean pre-sediment addition: 296 ± 52.8 stems m^−2^; mean post: 90 ± 26.2 stems m^−2^) and biomass (mean pre: 231 ± 85.3 g m^−2^; mean post: 94 ± 25.5 g m^−2^) every year after sediment placement at the impact site, but no change in stem density (mean pre: 247 ± 66.0 stems m^−2^; mean post: 249 ± 83.7 stems m^−2^) or biomass (mean pre: 153 ± 36.5 g m^−2^; mean post: 139 ± 59.8 g m^−2^) at the control site over time (Table 3). *D. spicata* stem density and biomass were significantly greater at the control site compared to the impact throughout the study. No differences in biomass or stem density were observed for *J. gerardii or S. patens*. There were more frequent observations of *P. australis* (presence/absence on transects) at the control site than the impact site regardless of year (logistic regression likelihood ratio test, *p* = 0.0003), and *P. australis* biomass and stem density were both significantly higher at the control site than the impact site throughout the study (Table 3).

#### Nekton

Nekton communities in the two sites were typical of southern New England marshes, with low species richness and a small number of highly-abundant species. The impact site supported 21 species from 2015 to 2019, dominated by C. *variegatus* (26% of the community based on density), F. *heteroclitus* (16%) and striped killifish (*F. majalis*) (12%); all other species comprised less than 10% of the community, with 10 species making up <1% (Supplementary Table S6). At the control site, 15 species were found and only two dominated (daggerblade grass shrimp *Palaemonetes pugio* 52% and *C. variegatus* 24%).

Nekton communities were significantly different between sites (PERMANOVA, *pseudo-F* = 38.11, *p* = 0.001) and over time (*pseudo-F* = 7.25, *p* = 0.001). There was also a significant site × time interaction (*pseudo-F* = 2.27, *p* = 0.004) with contrasts indicating that impact marsh nekton communities were significantly different every year after sediment placement compared to before, but not different between any pair of years after sediment placement (Supplementary Table S4). Nekton communities in the control marsh were different between every pair of years tested. Thus, using a BACI design, an immediate nekton community response to sediment placement activities was not detected, but the lack of change in nekton at the impact site over time after sediment placement, even while changes occurred each year at the control, indicates reduced interannual variability in nekton composition after sediment placement. From SIMPER, *F. heteroclitus* and *C. variegatus* typified the impact marsh before sediment placement, making up 77% of community similarity (Supplementary Table S7). After sediment placement, some combination of *F. heteroclitus*, *F. majalis*, green crab *Carcinus maenas*, long-armed hermit crab *Pagurus longicarpus*, and Atlantic silverside *Menidia menidia* typified the impact marsh each year, contributing a combined >70% to community similarity (*C. variegatus* did not contribute any year after sediment placement). These changes indicate an increased prevalence of sand-associated species in the impact marsh after sediment placement. The control site, in contrast, was consistently typified by marsh interior and brackish species such as *F. heteroclitus*, *C. variegatus*, *P. pugio* and rainwater killifish *Lucania parva*.

**FIGURE 8:**
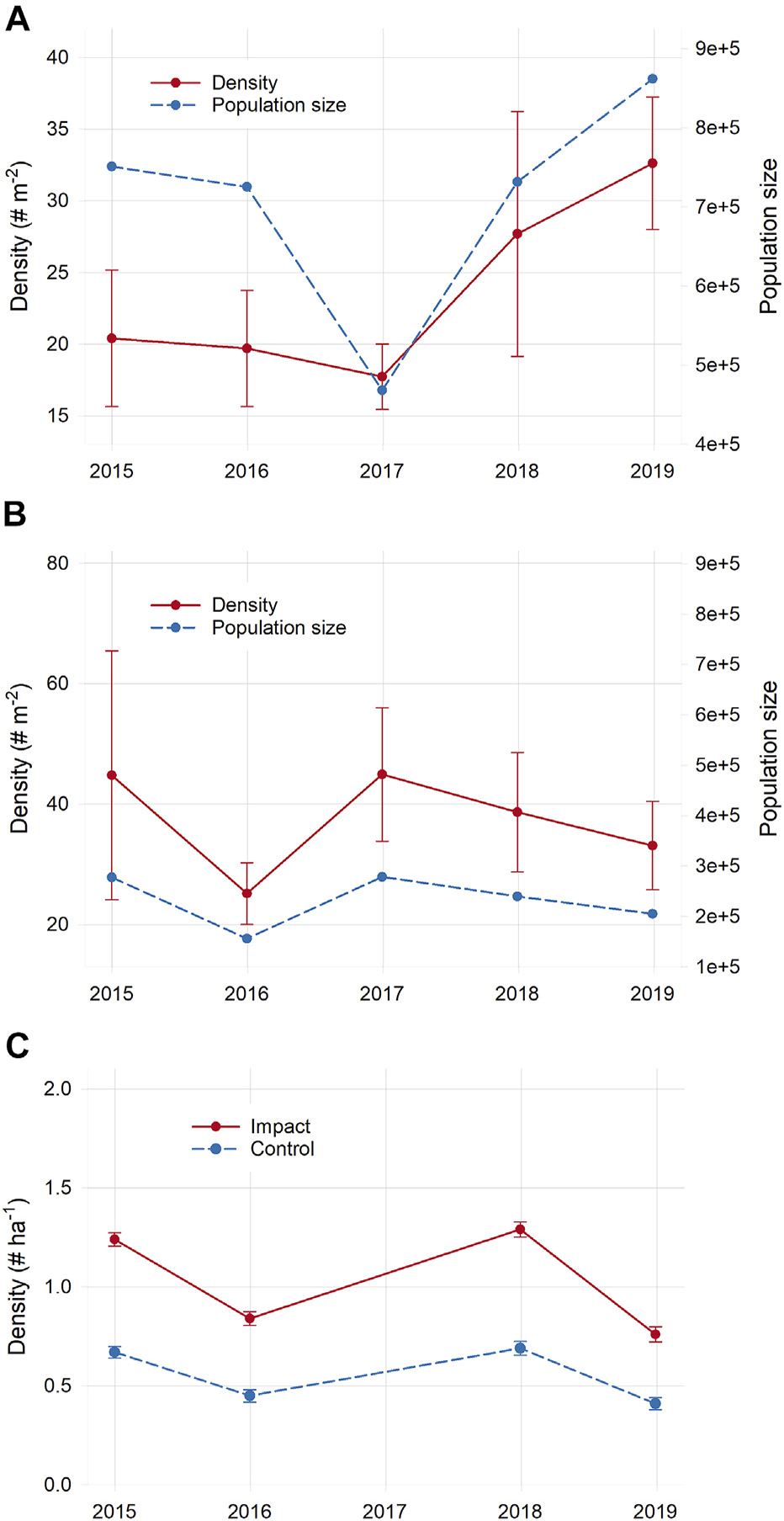
Changes in total nekton density and total nekton population size in the impact **(A)** and control **(B)** marshes, and total bird density **(C)** over time. Sediment placement occurred between 2016 and 2017 at the impact site. Error bars for density are 1 SE. Population size for each marsh × year was calculated by multiplying total density by the total area (in square meters) of all shallow aquatic habitat available to nekton in each marsh.

Mean total nekton density (all species combined) declined slightly (12%) the first year after sediment placement at the impact site, but rebounded in year two and exceeded initial levels by 63% in year three; the same pattern was found for total nekton population size, although the decline after sediment placement was more pronounced. Control marsh nekton density varied among years, but there was no clear trend over time (Figures 8A,B). These changes, however, were not statistically significant (Supplementary Table S8). For individual species, *C. variegatus*, *F. heteroclitus*, and *L. parva* densities declined in the impact marsh the first year after sediment placement, while the sand-associated species *F. majalis* and sand shrimp (*Crangon septemspinosa*) both increased. By year three post-sediment placement, densities of these species were either comparable or higher than initial. As with total density, however, significant site × time interactions were not detected for any individual species tested (except inland silverside *Menidia beryllina*, although there were no subsequent significant pairwise comparisons for this species) further indicating that sediment placement had no apparent effect on nekton density (Supplementary Table S8).

Changes in burrow density over time were nearly identical in both marshes, and there was no significant site × time interaction detected during our study, indicating that burrow density was apparently not affected by sediment placement (Supplementary Figure S2 and Supplementary Table S8).

#### Birds

Fifty-one bird species were identified during this study (not counting a small number of additional birds that could not be identified to species), 38 at the impact site and 45 in the control (Supplementary Table S9). The number of species at the impact site dipped to 20 species in 2018 when the marsh remained mostly bare compared to before sediment placement (28 species) and 3 years after (26 species). Red-winged Blackbird (*Agelaius phoeniceus*) was the most dominant species (highest mean density) at both sites, and other dominant species were generally common to both marshes, including Song Sparrow (*Melospiza melodia*), Willet (*Tringa semipalmata*), Saltmarsh Sparrow (*Ammospiza caudacuta*), and Mourning Dove (*Zenaida macroura*).

Bird communities were significantly different over time (PERMANOVA, *pseudo-F* = 4.20, *p* = 0.001) but not between sites (*pseudo-F* = 1.30, *p* = 0.20) and there was no significant site × time interaction (*pseudo-F* = 1.27, *p* = 0.16). Communities were significantly different between every pair of years tested in both sites, suggesting that there were no effects from sediment placement on bird community structure and changes were likely rather due to broader-scale interannual variability (Supplementary Table S4). As with nekton, there were no significant site × time interactions for total bird abundance (sum of counts of all species), nor for abundances of common species, indicating that sediment placement did not have an effect on bird abundance (Supplementary Table S8). Changes in total bird density and densities of guilds followed the same pattern across years in both marshes, suggesting that temporal variability was due more to broader-scale factors rather than localized sediment placement (Figure 8 and Supplementary Figure S3).

## Discussion

The term “thin-layer sediment placement” suggests to managers seeking to increase marsh resilience that they should act conservatively, by incrementally adding thin layers of sediment to degraded or drowning marshes. With this approach, the marsh system can be shifted to an optimal elevation for the low marsh dominant, tall-form *S. alterniflora*, to grow and persist, and there is evidence to support this strategy (Davis et al., 2022; Puchkoff and Lawrence, 2022). Adding a thin layer rather than a thick layer of sediment requires less dredge material for large-scale projects, but trade-offs include a smaller increase in elevation capital and higher long-term maintenance costs. In contrast, new evidence shows that in certain conditions adding thicker sediment layers can also be effective. In a study spanning marshes in eight U.S. estuaries, vegetation recovered more quickly after thin (7 cm) vs. thick (14 cm) additions of sandy quarry sediment, but after 3 years plant cover did not differ between the two thicknesses (Raposa et al., in review). But even the thicker addition (i.e., 14 cm) was ineffective at restoring high marsh plant species affected by SLR. Our study goes further, demonstrating that a marsh can largely revegetate with high marsh grass species, forbs, and herbs in just a few years after large-scale placement of very thick (30 cm+) sandy dredged sediment. Thus, placing a thick layer of clean, sandy sediments on the existing marsh platform might be the preferred approach if the goal is to maximize elevation capital and shift plant dominance from tall-form *S. alterniflora* to high marsh species such as *S. patens* and *J. gerardii*. We caution, however, that adding thick sediment layers may only be appropriate when using sand-dominated sediments; thicker layers of silt and clay-dominated sediments, particularly acid sulfate soils, may hinder revegetation due to oxidation of iron sulfides (Berkowitz and VanZomeren, 2020; Puchkoff and Lawrence, 2022).

One of the advantages of adding a thick sediment layer is that it can be cost-effective. In our study, the net cost of adding 10–48 cm of sediment to the marsh (after subtracting the ~ $1M cost of dredging, which the town planned to complete whether or not material was used for marsh restoration) was approximately $600,000 (inclusive of adaptive management activities). Typically, these dredged sediments would have been used for beach nourishment, but in this restoration project they were instead used to increase elevation of the drowning Ninigret marsh. Assuming an ambient rate of elevation gain of 1.3 mm yr^−1^ (the rate from the control site during this study, similar to the 1.5 mm yr^−1^ mean rate of RI marshes; Raposa et al., 2017b) and the 10–48 cm of sand added, this project resulted in an estimated 67–320 years of elevation gain in just one winter at relatively small cost, without the need for multiple applications of thinner sediment layers over time.

### Vegetation

Sediment placement successfully increased elevation capital for foundational high marsh plant species at the impact site, and thus increased salt marsh resilience. Unlike some restoration efforts targeting platform elevations that just meet optimal low marsh vegetation elevations and require only thin-layer sediment placement, project stakeholders decided to aim for the upper end of high marsh plant elevation growth ranges to build elevation capital and minimize maintenance costs of additional sediment applications. Consequently, recovery to optimal growth and biomass may take longer for some plant species, especially tall-form *S. alterniflora*, the dominant low marsh species, but the recovery trajectory evident from time-series and community analyses, which includes all species, is trending positive.

We caution that targeting high elevations to maximize elevation capital and promote optimal growth conditions for high marsh graminoids might inadvertently promote invasive species such as *P. australis.* The control site had higher *P. australis* cover in monitoring plots compared to the impact site throughout the study. However, *P. australis* elsewhere in the impact site was managed throughout the study by restoration specialists who were engaged in *P. australis* removal *via* hand-pulling and hydrological interventions (e.g., runnels). These management actions may have helped limit *P. australis* colonization into monitoring plots at the impact site; future monitoring after cessation of invasive species management will be needed to ultimately document *P. australis* response to adding sediment targeted at the upper end of optimal elevation ranges. More optimistically, these higher-elevation areas were rapidly colonized by ecotone species after sediment placement and should serve as future marsh migration corridors as sea-level rise continues.

Under more natural conditions, bare areas on marsh platforms might result from wrack deposition (Bertness and Ellison, 1987), but in this study large swaths of bare marsh were created by sediment placement. In natural bare areas, hypersaline conditions often restrict typical marsh grass dominants such as *S. patens* and promote seed-bearing, somewhat or fully succulent, annual colonizers such as *Salicornia* spp., forbs, and herbs. Subsequently, these early colonizers provide shade, reduce hypersaline conditions, and promote vegetative propagation by marsh grasses such as *D. spicata* and *S. patens* (Bertness et al., 1992; Crain et al., 2008). In our study, we noticed colonization of bare areas at the impact site by successional colonists (*S. marina*, *Suaeda* spp., *Atriplex* spp. and *Salicornia* spp.) in 2018–2020. We suspect that in future years, just as was reported for recovering bare spots due to wrack deposition (Bertness and Ellison, 1987), natural succession will lead to subsequent dominance of marsh graminoids (e.g., *D. spicata*, *J. gerardii*, *S. patens*) in areas where early colonizers are presently common.

Placement of sediment on the impact site caused an increase in bare ground cover the first 2 years following placement, but by years three (2019) and four (2020), marsh plants had successfully re-vegetated bare areas to pre-placement and control levels. Similarly, the combined cover of the dominant two plant species at the impact site was not different from pre-sediment placement or control site cover by year three. Although we did not observe significant differences in *Spartina* species cover between the impact and control sites in 2019 or 2020, SIMPER and trend analyses suggested that while high marsh graminoid cover at the impact site exhibited a clear upward trajectory after sediment placement, cover at the control site remained stable. These community analyses also accounted for less-common plant species such as saltworts, forbs, and herbs, which were conspicuous colonizers of high-elevation bare areas at the impact site and demonstrated plant community succession as the marsh recovered.

Stem density and biomass of tall-form *S. alterniflora* were significantly lower every year after sediment placement at the impact site compared to before, but were stable over time at the control site, suggesting this low-marsh dominant species was still recovering at the impact site 4 years after sediment placement. There were no apparent effects of sediment placement on stem density and biomass of other common species at the impact site. Future monitoring will determine if the elevation capital gains at the impact site will facilitate further increases in cover, stem density, and biomass of dominant grass species (*S. alterniflora, S. patens*, *D. spicata*, *J. gerardii*) and whether plant cover at the control site remains stable or begins to decline with expected accelerated SLR.

### Fauna (nekton and birds)

To our knowledge, ours was the first study to assess nekton and bird responses to sediment placement in the northeast U.S. For nekton, previous studies evaluating effects of marsh creation using dredged material in the Gulf of Mexico show that created marshes, regardless of age, provide valuable habitat for nekton (Minello and Webb, 1997). Results from our study are similar; the only detectable effect of sediment placement on nekton was a consistent prevalence of sand-associated species after placement; densities of individual species and total nekton were unaffected. These findings are likely due in part to aspects of project design as well as our chosen sampling method. By design, sediment was placed on just 8.9 ha within the entire 15-ha marsh study area–the remaining ~6 ha of existing marsh vegetation was left available for nekton to use for possible foraging, refuge, and spawning during high tides (Allen et al., 1994; Kneib, 1997). We also decided to sample nekton with throw traps, which are widely used in part because they have very high capture efficiencies when used in shallow aquatic habitats such as channels, creeks, and natural pools (Raposa and Talley, 2012). These habitats were also specifically avoided when placing sediment during this project because they were identified as already providing habitat for nekton (Fuss & O’Neill, 2015). Perhaps significant impacts on nekton would have been identified had we chosen to also sample in locations on the marsh surface before and after sediment placement with bottomless lift nets or other appropriate gear (Rozas, 1992; Rozas and Minello, 1997), or if sediment placement occurred on a much greater proportion of the study site. At a minimum, our study provides new evidence that a thick layer of sediment can be added to large portions of deteriorating northeast U.S. marshes with likely very minimal and short-term effects on nekton communities.

Similarly, bird composition and abundance were unaffected by sediment placement, though this is unsurprising given the spatial and temporal scale of the project. It seems clear that highly-mobile birds were responding to factors beyond the scale of our study site given the matching temporal patterns observed between the impact and nearby control marsh. Previous studies demonstrate that birds readily use new marsh created from dredged material (Melvin and Webb, 1998; Darnell and Smith, 2004) and other types of restored marshes, sometimes at comparable or greater levels than reference marshes (Brawley et al., 1998; Seigel et al., 2005). Results from our study are similar, showing that birds used the impact marsh even during the first year after sediment placement when it was largely bare ground. Although, as with nekton, perhaps setting aside 40% of the total study site created a diverse habitat mosaic favorable to a rich and abundant bird community. While the structure of bird communities in the impact marsh was unaffected by sediment placement, our study was not designed to assess possible changes in the way the marsh functioned as habitat for birds. We therefore recommend that future studies include an avian demographic monitoring component to better understand how sediment placement affects the ways various bird species use marshes for foraging, nesting and as cover.

We do note, however, some anecdotal species-specific observations after sediment placement that may warrant further study in future projects. After sediment placement (2018 and 2019), we detected Piping Plovers (*Charadrius melodus*) in the impact site for the first time and detected twice as many Least Terns (*Sternula antillarum*) in the impact site in 2018 compared to any other year, though this difference was not significant. Beach nesting birds such as these are drawn to the bare sand deposited on the marsh surface, and we recommend that future projects consider the potential short-term benefits of large-scale sediment placement on these species. In addition, the Saltmarsh Sparrow (*A. caudacuta)*, which breeds exclusively in northeast U.S. salt marshes, nests in high marsh vegetation, and is in a steep 9% annual decline (Correll et al., 2017), was unaffected by sediment placement. It is encouraging for future sediment placement projects to note that this species was not locally extirpated during our project and can potentially respond quickly when high marsh vegetation recovers.

### Adaptive management

Adaptive management can be an essential component of successful restoration projects (Buchsbaum and Wigand, 2012; Perry et al., 2020), but to what extent did adaptive management activities of the landscape and hydrology facilitate some of the outcomes documented after sediment placement in this study? We observed, but did not directly quantify, some apparent benefits (Supplementary Figure S4). For example, *P. australis* was heavily managed after sediment addition, in part by coupling additional grading with the creation of runnels to facilitate drainage of impounded groundwater and precipitation from higher-elevation areas at the upper edge of the sediment placement area. *Phragmites australis* was also spot treated in some locations at the impact site (never on the monitoring transects) with herbicide by a licensed applicator during the first year and then pulled by hand in subsequent years. These adaptive management activities helped limit the cover and biomass of this invasive species at the impact site, at least initially. Additional runnels that were dug through the salt marsh sill at the lower edge of the sediment placement area were effective at draining new shallow ponded areas and, apparently, reducing pore water salinity levels that were preventing plant re-colonization due to hypersaline conditions. The runnels also served as a conduit for fish passage.

Plantings quickly established thousands of new marsh plant seedlings after sediment placement, and we observed rapid vegetative spread within planted patches and sediment accumulation around new plants (note that we did not directly quantify survival rates of different planted species, but we generally achieved the highest survival with *D. spicata*, likely due to its high salinity tolerance, while *S. alterniflora* survival was variable, with poor results at lower elevations where anoxic soils were present during the first growing season; *S. patens* and *J. gerardii* were generally difficult to establish). We also observed natural colonization of *S. patens* and *J. gerardii via* seeds from existing plants adjacent to the sediment placement area, suggesting that natural colonization may be important for large-scale revegetation of some species. Coupled with evidence that thick layers of added sediment can limit vegetative regrowth of buried plants (Mendelssohn and Kuhn, 2003), this highlights the possible benefits to leaving some areas of thick sediment placement projects intact to act as a local seed source for desired species. We did not examine how different mechanisms contributed to overall vegetation recovery, but it seems clear that plantings and natural recolonization from existing plants both facilitated early recovery in our study. Given the cost and effort involved with extensive planting, future work should be designed to quantify its benefits locally within planted areas and more broadly across entire sediment placement marsh landscapes.

In future sediment placement restoration projects, we recommend embedding experiments (Gellie et al., 2018) to better understand natural colonization rates, planting success rates, effects of restoring hydrology through creek and runnel establishment, and other adaptive management activities. Our restoration monitoring plan was not designed to disentangle the effects of all management activities, but we are confident that adaptive management of the landscape and hydrology provided benefits to the initial sediment placement, at least in the short-term and/or at small-scales.

### Restoration trajectory

Some study findings should be considered preliminary because only four growing seasons of monitoring occurred after sediment addition. Physical changes were rapid and clear, including increased elevation capital, marsh surface stabilization, and improved marsh drainage. In contrast, soil characteristics (% organic matter, % moisture, and bulk density) may take a decade or more to reach equivalency with the control marsh and other reference salt marshes in southern New England (Warren et al., 2002; Craft et al., 2003). Some vegetation and landscape changes may still be underway. For example, some of the higher-elevation areas readily colonized with upland ecotone and upper marsh edge species (*B. halimifolia, S. sempervirens, P. virgatum*, and *Morella pensylvanica*) soon after sediment placement (Supplementary Table S3), establishing early migration corridors and setting the stage for possible future landward marsh migration. Nekton density, bird abundance, and bird community structure appear to be unaffected by sediment placement in the short-term; however, as impact site vegetation fully recovers, as suggested by the ongoing positive trajectory of high marsh species, there may be corresponding responses in some fauna. For example, less inundation of the high marsh achieved by the increase in elevation capital, and the recovery of high marsh *S. patens*, may facilitate recovery of the threatened Saltmarsh Sparrow. Biological and edaphic responses to marsh restoration often take longer to develop than physical ones (Konisky et al., 2006; Raposa et al., 2018); for future sediment placement projects, we therefore recommend more than 3 years of monitoring after sediment addition, or a few years immediately after, followed by future intermittent snapshot assessments (e.g., every 3–5 years, for at least one decade) to document both short and long-term change.

## Conclusion

Measured against the two *a priori* goals, this project was a partial success after four years–the goal of increasing marsh resilience to SLR was quickly achieved, but it was not yet apparent if the goal of restoring high marsh habitat was fully met. Measured against objectives common across sediment placement projects (Raposa et al., 2020), however, this project was broadly successful. Proper elevations were achieved, resilience increased, the sediment placement area revegetated rapidly and shifted towards a high marsh-dominated community, and bird communities were indistinct from the control site. For our study marsh, a thick addition of dredged sandy sediment was deemed most-appropriate to achieve project goals, and this approach might be best for future projects where the goals are to restore high marsh species, build elevation capital, and establish a migration corridor for saltmarsh habitat. For any sediment placement project, determining the targeted range of elevations and the corresponding thickness of added sediment is one of the most important aspects of project design and it ultimately depends on many factors including added sediment type, tide range, existing marsh elevation and plant distributions, project goals and desired lifespan of the new marsh, as well as invasives. Our study demonstrates that adding a thick layer of sandy sediment is a viable restoration option, but note that adding thinner layers may be a more appropriate option under certain conditions.

## Supplementary Material

Supplement1

## Figures and Tables

**TABLE 1:** Monitoring parameters included in this study.

Category	Method	Parameter(s)	2014	2015	2016	2017	2018	2019	2020

Photography	Photo stations	Visual time series of marsh change		I	I	I	I	I	I
Elevation	RTK	Marsh elevation	I			I	C/I		
	SETs	Net elevation change		C/I	C/I	C/I	I	I	C/I
	Marker horizons	Accretion, subsidence					I		
Hydrology	HOBO logger in pond	Tidal datums					C/I		
	HOBO loggers in marsh	Marsh surface inundation and drainage		C/I	C/I	C/I	C/I	C/I	
	Salinity mapper	Marsh-wide salinity		C/I	C/I	C/I	C/I	C/I	
Soils	Shear vane	Shear strength		C/I	C/I	C/I	C/I	C/I	
	Soil cores	% moisture, bulk density, % organic matter, belowground biomass		C/I				C/I	
Vegetation	Plots	Community composition, cover, height, stem density		C/I	C/I	C/I	C/I	C/I	C/I
	Aboveground biomass	Biomass		C/I	C/I	C/I	C/I	C/I	C/I
Nekton	Throw traps	Community composition, density		C/I	C/I	C/I	C/I	C/I	
	Burrow counts	Crab burrow density		C/I	C/I	C/I	C/I	C/I	
Birds	SHARP surveys	Community composition, density		C/I	C/I		C/I	C/I	

Years before sediment placement are 2015–2016; years after are 2017–2020. C, control site; I, impact site.

**Table 2:** Two-factor ANOVA main effects and site × time interactions examining vegetated and unvegetated cover (as indicated), using transect mean percent cover values.

Cover type	Site × time interaction	Site difference	Time difference	Site comparison	Time comparison

All vegetation (sum)	Yes (*p* = 0.0108)	N/A (Int.)	N/A (Int.)	2017: C>I	I: Pre>2017
High marsh graminoids	No (*p* = 0.2647)	No (*p* = 0.2330)	No (*p* = 0.2787)	N/A	N/A
Distichlis spicata	No (*p* = 0.8431)	Yes (*p* = 0.0002)	No (*p* = 0.8538)	C>I	N/A
Juncus gerardii	No (*p* = 0.6425)	No (*p* = 0.8618)	No (*p* = 0.4293)	N/A	N/A
Spartina patens	No (*p* = 0.0943)	No (*p* = 0.2228)	No (*p* = 0.1691)	N/A	N/A
Dominant two species	Yes (*p* = 0.0185)	N/A (Int.)	N/A (Int.)	2017: C>I	I: 2017, 2018<Pre
Successional colonists*	Yes (*p* = 0.0071)	N/A (Int.)	N/A (Int.)	2019, 2020: I>C	I: 2019, 2020>Pre
Salicornia depressa	No (*p* = 0.9923)	No (*p* = 0.4287)	No (*p* = 0.2982)	N/A	N/A
Spartina alterniflora	No (*p* = 0.1730)	No (*p* = 0.0784)	Yes (*p* = 0.0138)	N/A	Pre>2017
Phragmites australis	No (*p* = 0.9426)	Yes (*p* < 0.0001)	No (*p* = 0.9971)	C>I	N/A
Unvegetated	Yes (*p* = 0.0002)	N/A (Int.)	N/A (Int.)	2017, 2018: I>C	I: 2017, 2018>Pre
Bare ground	Yes (*p* < 0.0001)	N/A (Int.)	N/A (Int.)	2017, 2018: I>C	I: 2017, 2018>Pre
Open water	No (*p* = 0.9007)	Yes (*p* = 0.0013)	No (*p* = 0.9647)	C>I	N/A

Significant results are highlighted in green.

**TABLE 3:** Two-factor ANOVA main effects and site × time interactions examining stem count and estimated biomass for species (as indicated), using transect mean percent cover values.

	Species	Site × time interaction	Site difference	Time difference	Site comparison	Time comparison

Stem Density	Distichlis spicata	No (*p* = 0.6482)	Yes (*p* = 0.0005)	No (*p* = 0.6952)	C>I	N/A
	Juncus gerardii	No (*p* = 0.5551)	No (*p* = 0.6803)	No (*p* = 0.3420)	N/A	N/A
	Phragmites australis	No (*p* = 0.4032)	Yes (*p* < 0.0001)	No (*p* = 0.6097)	C>I	N/A
	Spartina alterniflora	Yes (*p* = 0.0036)	N/A (Int.)	N/A (Int.)	N/A	I: Pre>each post year
	Spartina patens	No (*p* = 0.2584)	No (*p* = 0.2173)	No (*p* = 0.2594)	N/A	N/A
Biomass	Distichlis spicata	No (*p* = 0.6366)	Yes (*p* = 0.0008)	No (*p* = 0.6566)	C>I	N/A
	Juncus gerardii	No (*p* = 0.4497)	No (*p* = 0.6289)	No (*p* = 0.3797)	N/A	N/A
	Phragmites australis	No (*p* = 0.4499)	Yes (*p* < 0.0001)	No (*p* = 0.6962)	C>I	N/A
	Spartina alterniflora	Yes (*p* = 0.0117)	N/A (Int.)	N/A (Int.)	N/A	I: Pre>each post year
	Spartina patens	No (*p* = 0.2417)	No (*p* = 0.1141)	No (*p* = 0.2664)	N/A	N/A

## Data Availability

The raw data supporting the conclusions of this article will be made available by the authors, without undue reservation.
