# Supplementary material for: Laying it on thick: Ecosystem effects of sediment placement on a microtidal Rhode Island salt marsh
Source: Front Environ Sci. Author manuscript; Available in PMC 2023 Sep 6. (PMC9728635; doi:10.3389/fenvs.2022.939870)

**SUPPLEMENTARY MATERIAL**

**Supplementary Figure 1.** Salinity mapping at the Ninigret impact and control sites. Top four panels: Contour plots of calculated salinity across the marsh surface of the control (a and c) and impact (b and d) marshes generated using inverse distance weighted interpolation from data collected before sediment placement in July 2015 and September 2016. Bottom six panels: Contour plots of calculated salinity across the marsh surface of the control (a, c, and e) and impact (b, d, and f) marshes generated using inverse distance weighted interpolation from data collected after sediment placement in September 2017, 2018, and 2019.

To create these salinity maps, we used a Geonics Model EM38-MK 2 Conductivity Meter (Geonics Ltd, Mississauga, Ontario, Canada) held 50 cm over the marsh surface to record apparent conductivity of the soil to a depth of 50 cm. The entire marsh platform was haphazardly surveyed by walking a zig-zag path with the conductivity meter and sampling an average of 147 ± 9.5 points at the impact and 80 ± 10.2 points at the control site over the course of the project. Apparent conductivity values in milliSeimans along with the latitude and longitude of the sample point and vegetation characteristics were entered into an ArcGIS shapefile using ArcPad software (ESRI, Redlands, CA) on a Trimble Nomad hand-held field computer (Trimble Navigation Ltd., Sunnyvale, CA USA). Both the impact and control marshes were surveyed annually during the growing season from 2015 through 2019; data collection and analysis followed the methods described in McKinney et al. (2019).

Mapping results in the figures below show that mean whole-marsh calculated salinity from 2015 to 2019 ranged from 19.9 ± 9.66 to 36.6 ± 7.86 ppt at the impact site and from 18.7 ± 5.98 to 29.8 ± 5.26 ppt at the control site; both were highest in 2016. Mean calculated salinity at the impact site showed a decreasing trend after sediment placement and differed among years (ANOVA: *df* = 4, *F* = 54.0, *P* < 0.001); the value in 2019 (20.2 ± 9.39 ppt) was significantly lower than in 2018 (25.1 ± 10.3 ppt) and 2017 (27.0 ± 10.9 ppt; Tukey-Kramer, *P* < 0.05). A similar trend was observed at the control site (ANOVA: *df* = 4, *F* = 54.0, *P* < 0.001; Tukey-Kramer, *P* < 0.05).

**References**

McKinney RA, Hanson AR, Johnson R, Charpentier M (2019) Seasonal variation in apparent conductivity and soil salinity at two Narragansett Bay, RI salt marshes. *PeerJ* 7:e8074 <http://doi.org/10.7717/peerj.8074>


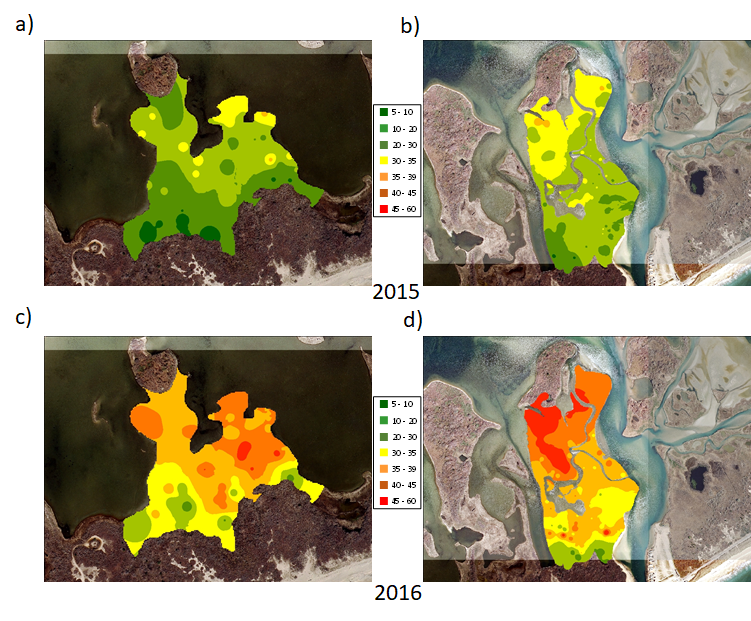


**
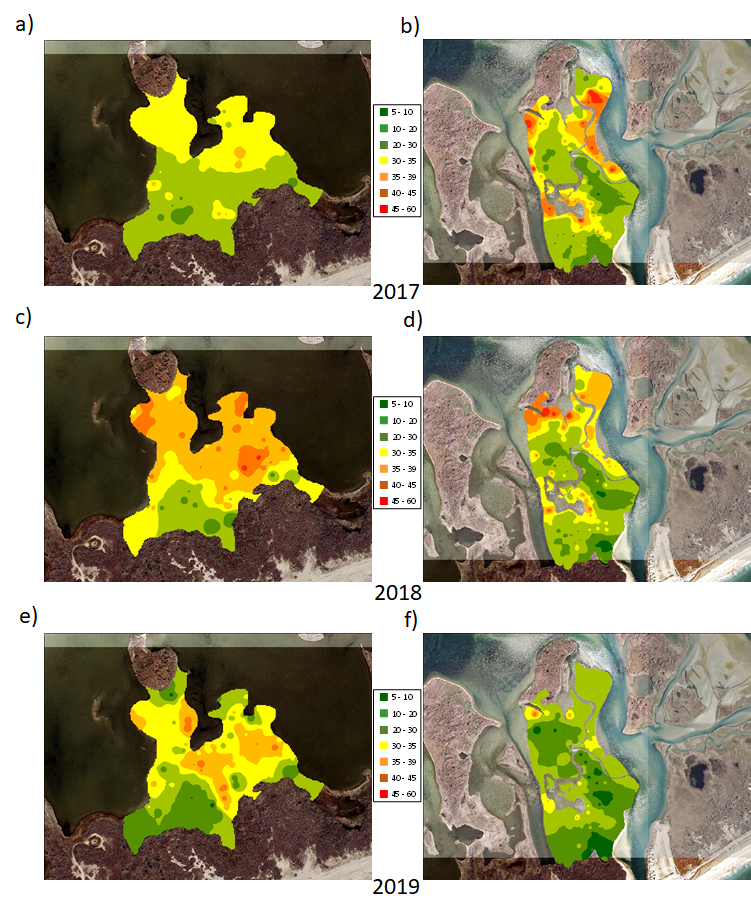
**

**Supplementary Table 1.** Best-fit equations used to estimate stem biomass (in equations y = stem biomass and x = mean stem length). Aboveground biomass for each species was estimated as stem density multiplied by stem biomass.


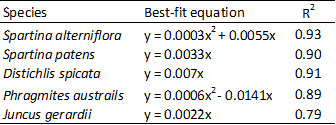


**Supplementary Table 2**. Three-factor interactions and main effects of site, year, and depth reported for soil moisture fraction, bulk density, and fraction of organic matter. Differences in roots were described by a two-factor model (site x year) as indicated.


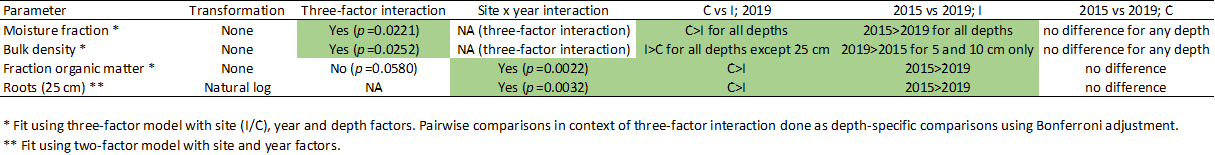


**Supplementary Table 3.** Cover of vegetation species in the impact (top) and control (bottom) marshes over time. Data for each year are means and 1 SE from replicate quadrats within each marsh. 2015 and 2016 are prior to sediment placement; 2017-2020 are after. Within each marsh, species are sorted in decreasing order based on mean overall cover across years, with bare and open water offset within unvegetated.

**
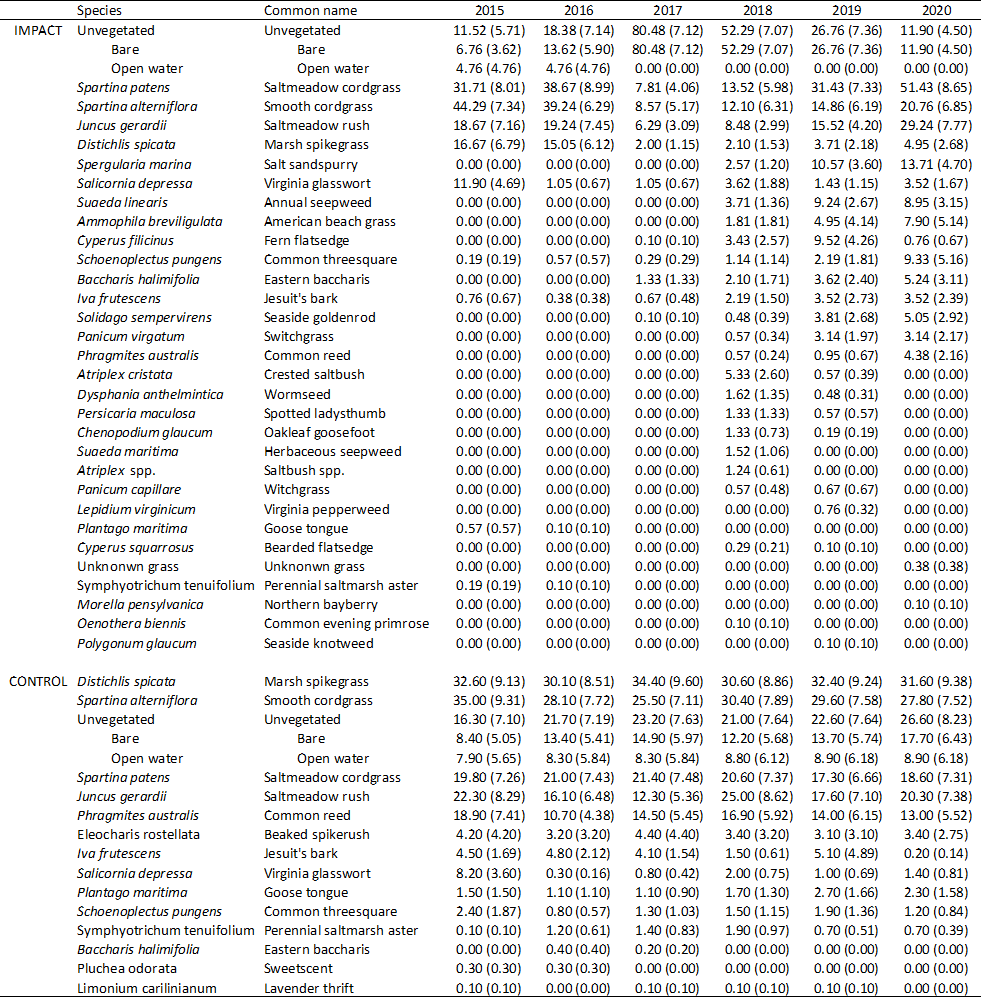
**

**Supplementary Table 4.** PERMANOVA custom contrast results in the impact and control marshes for vegetation (top), nekton (middle) and bird (bottom) communities. Pairs that are significantly different are highlighted in green.


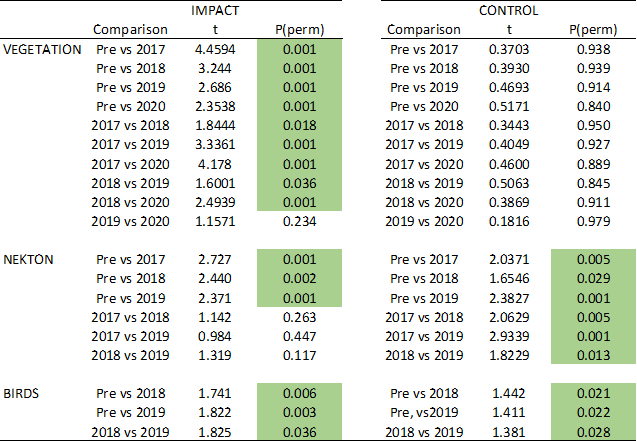


**Supplementary Table 5.** Results from similarity percentages (SIMPER) analysis of vegetation communities. For each year in each marsh, results are shown for species that collectively contributed >70% to cumulative overall similarity. Av. Sim = mean Bray-Curtis similarity between all pairs of stations (column 2) or contribution of individual species to overall mean similarity (column 5); Av.Abund = mean weighted cover; Sim/SD = mean similarity divided by standard deviation–species with higher values are consistent indicators of similarity for that marsh and year; Contrib% = percent contribution of individual species to overall similarity; Cum.% = cumulative sum of overall similarity.

**
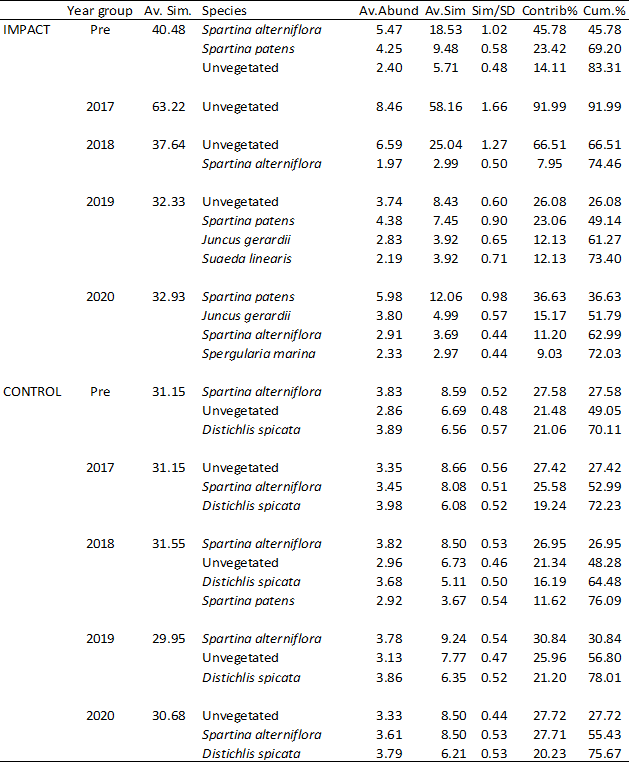
**

**Supplementary Table 6**. Nekton species densities in the impact (top) and control (bottom) marshes. Data for each year are means and 1 SE using each trap sample, averaged across months, as a single replicate. 2015 and 2016 are prior to sediment placement; 2017 to 2019 are after. In each marsh, species are sorted in decreasing order based on mean overall density across years.


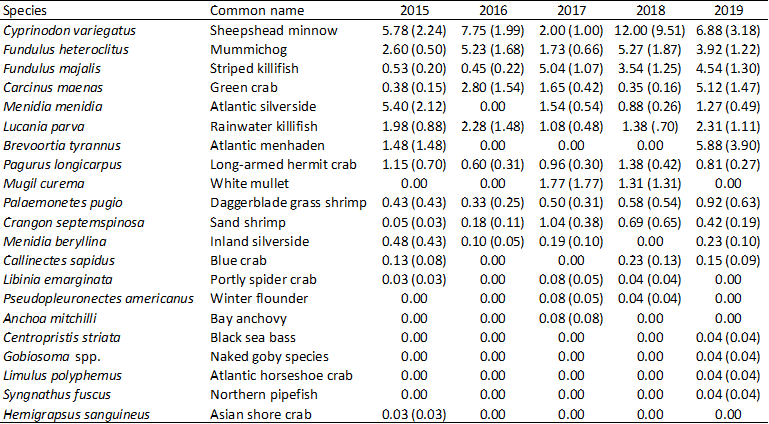


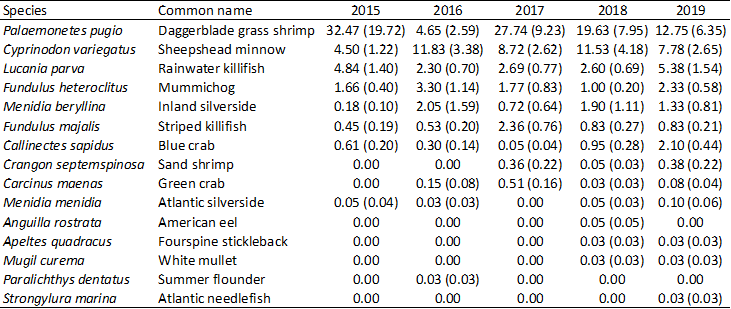


**Supplementary Table 7.** Results from similarity percentages (SIMPER) analysis of nekton communities. For each year in each marsh, results are shown for species that collectively contributed >70% to cumulative overall similarity. Av. Sim = mean Bray-Curtis similarity between all pairs of stations (column 2) or contribution of individual species to overall mean similarity (column 5); Av.Abund = mean weighted cover; Sim/SD = mean similarity divided by standard deviation–species with higher values are consistent indicators of similarity for that marsh and year; Contrib% = percent contribution of individual species to overall similarity; Cum.% = cumulative sum of overall similarity.


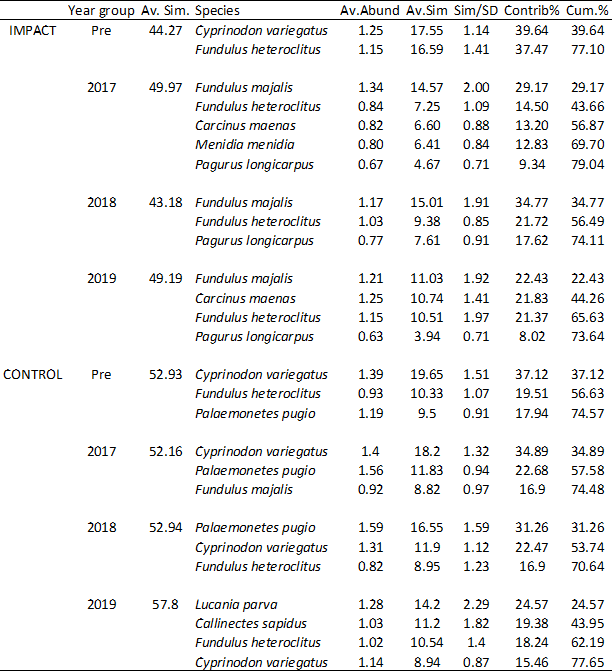


**Supplementary Table 8.** Statistical results from analyses comparing densities of nekton, crab burrows, and birds between sites and across years. Nekton and crab burrow densities were analyzed using logistic regression models with significance evaluated using Likelihood Ratio Chi-Square tests, except total nekton, *C. variegatus*, and *F. heteroclitus* densities, which were analyzed using two-factor ANOVA and Normal score transformation; bird densities were analyzed using two-factor ANOVA using log transformation and pairwise time comparisons conducted using Bonferroni correction. Significant results are highlighted in green.


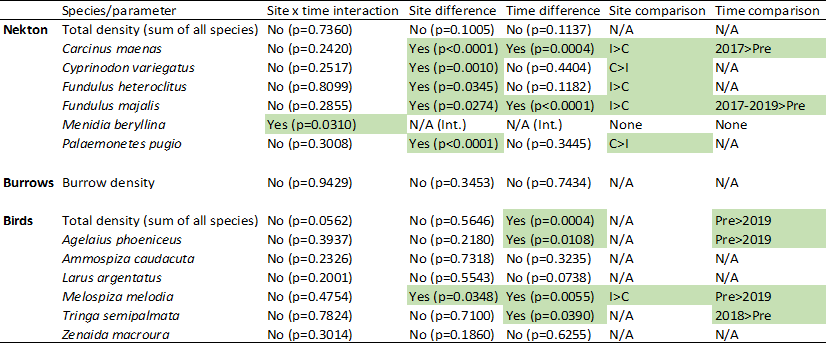


**Supplementary Figure 2**. Changes in crab burrow density over time in the impact and control marshes. Error bars are 1 SE.


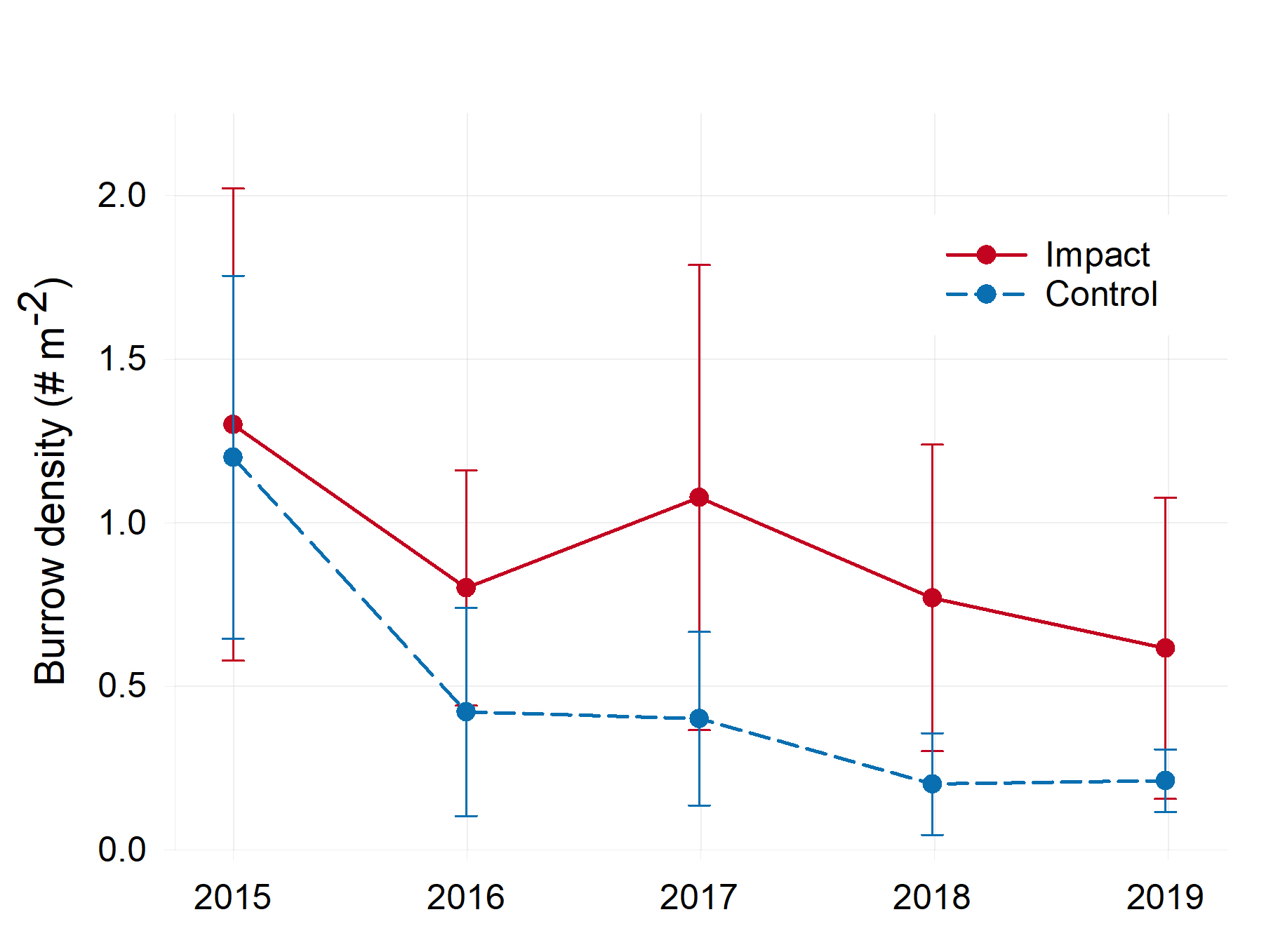


**Supplementary Table 9**. Avian species densities in the impact (left) and control (right) marshes over time. Sediment placement occurred in the impact marsh between 2016 and 2017 (no data were collected in 2017). Densities were estimated using package ‘unmarked’ in Program R. Parenthetical error terms are 1 SE; 'NA' indicates standard errors could not be estimated. ‘X’ indicates densities could not be estimated due to low occurrence. Species are sorted in descending order based on mean overall density in the impact marsh.

**
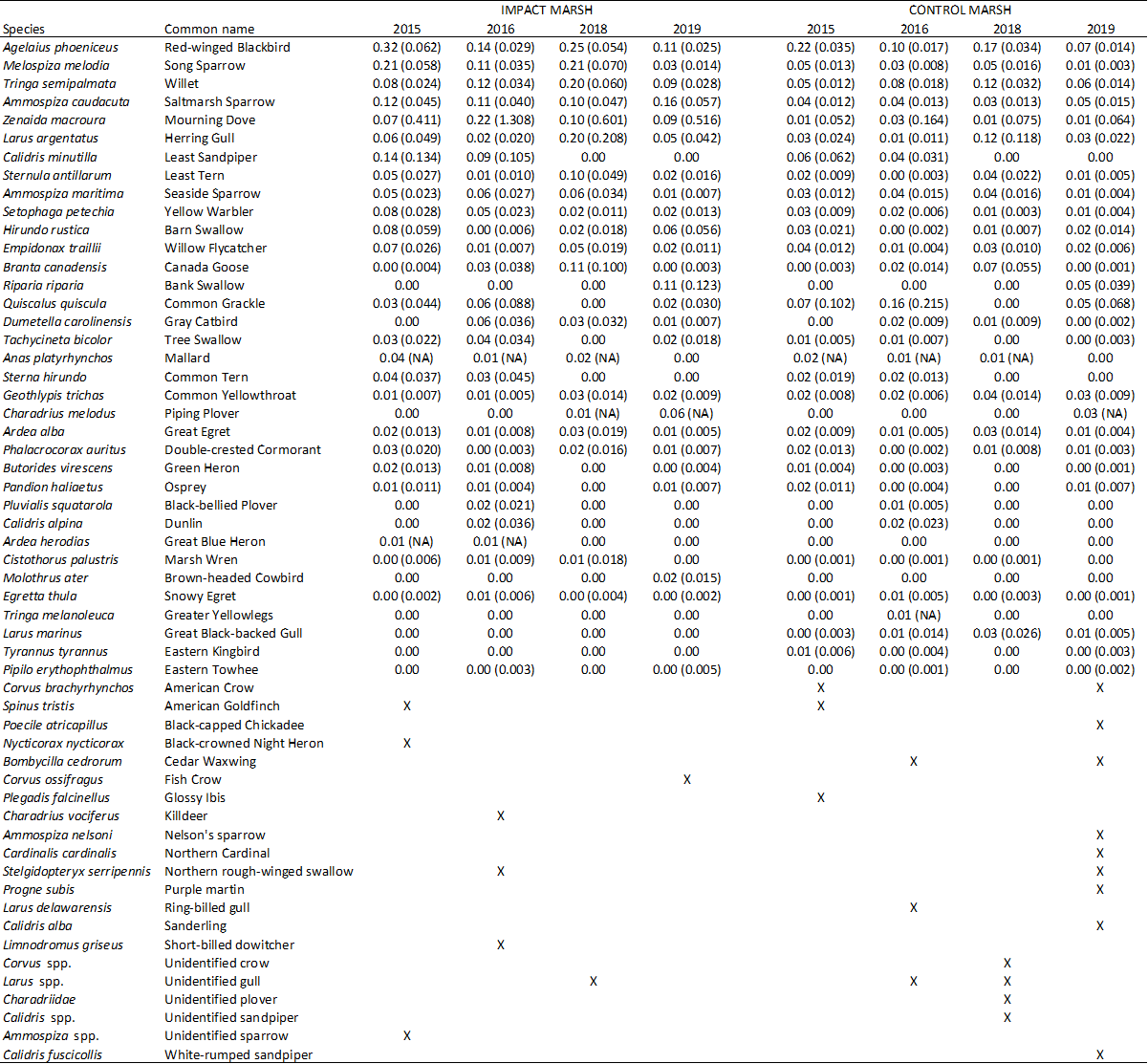
**

**Supplementary Figure 3**. Avian guild densities in the impact and control marshes over time. Sediment placement occurred in the impact marsh between 2016 and 2017 (no data were collected in 2017). Densities were estimated using package ‘unmarked’ in Program R. Error bars are 1 SE. Guild definitions provided at the bottom (for scientific names, see Supplementary Table 9).


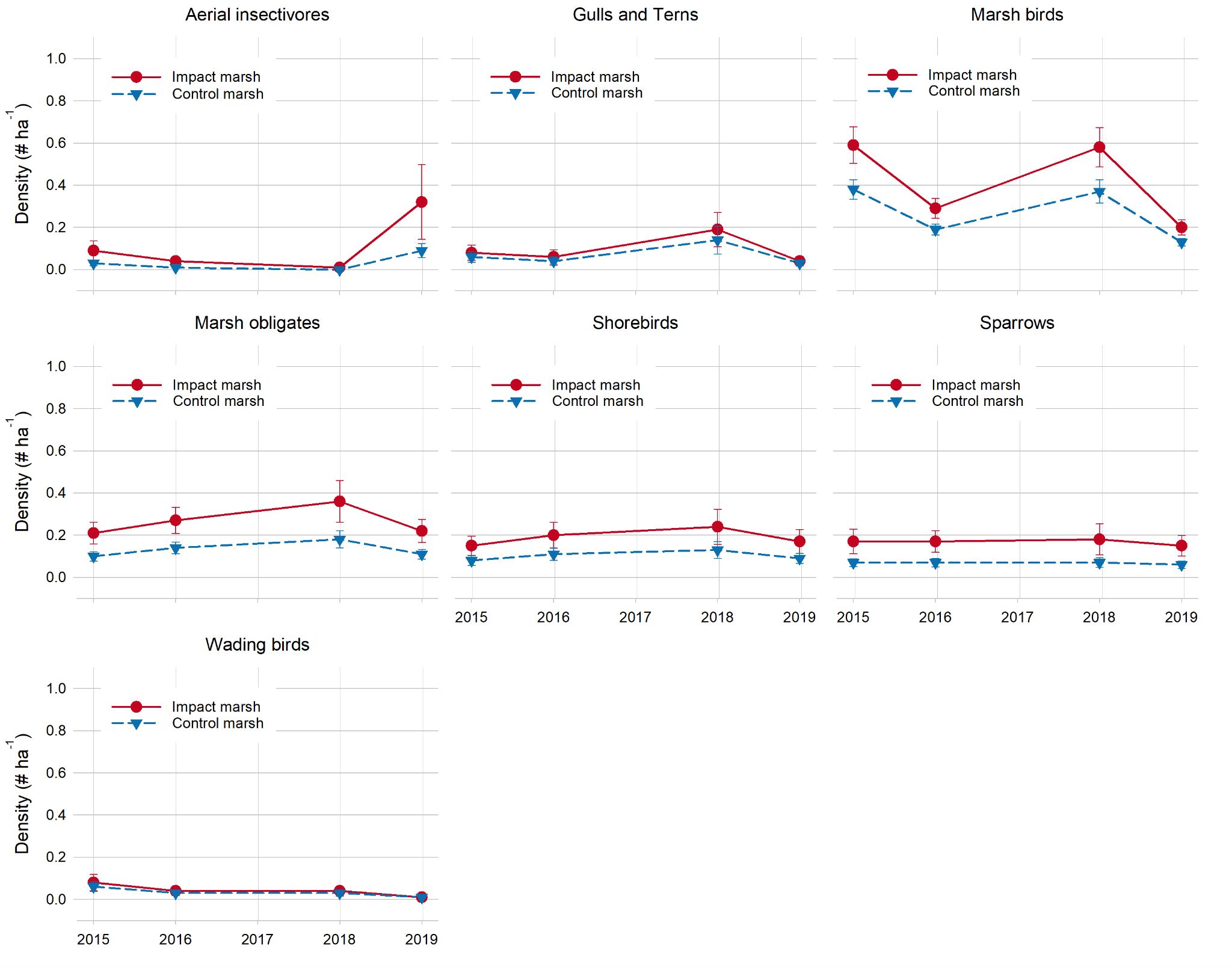


**Guild definitions**:

**Aerial Insectivores** = Bank Swallow, Barn Swallow, Northern Rough-winged Swallow, Purple Martin, Tree Swallow. **Gulls and Terns** = Common Tern, Great Black-backed Gull, Herring Gull, Least Tern, Ring-billed Gull, Unidentified Gull. **Marsh Birds** = Canada Goose, Common Grackle, Common Yellowthroat, Double-crested Cormorant, Mallard, Marsh Wren, Osprey, Red-winged Blackbird, Song Sparrow, Willow Flycatcher. **Shorebirds** = Black-bellied Plover, Dunlin, Greater Yellowlegs, Killdeer, Least Sandpiper, Piping Plover, Sanderling, Short-billed Dowitcher, Unidentified Plover, Unidentified Sandpiper, White-rumped Sandpiper, Willet. **Tidal Marsh Sparrows** = Nelson’s Sparrow, Saltmarsh Sparrow, Seaside Sparrow, Unidentified Sparrow. **Tidal Marsh Obligates** = Nelson’s Sparrow, Saltmarsh Sparrow, Seaside Sparrow, Willet. **Wading Birds** = Black-crowned Night Heron, Glossy Ibis, Great Blue Heron, Great Egret, Green Heron, Snowy Egret.

**Supplementary Figure 4.** Example pictures of adaptation management activities at the Ninigret sediment placement marsh. Grading and runnelling to facilitate drainage (A); creek excavation to drain impounded fresh weather (B); runnel through peat to address hypersalinity and drain surface water (C); hand-dug runnel to drain freshwater (D); hand-pulling invasive *Phragmites australis* (E); planting *Spartina alterniflora* plugs (F).


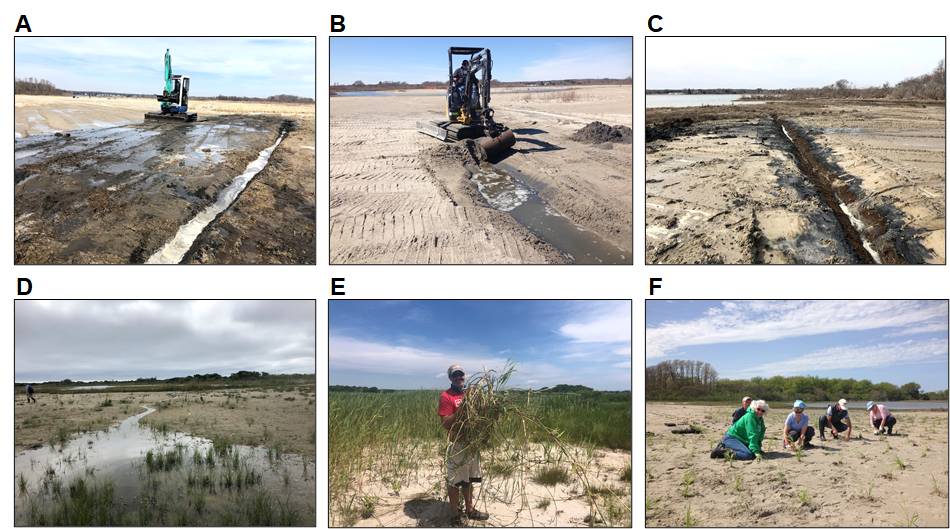

Supplement: Supplement1 [file NIHMS1848986-supplement-Supplement1.docx]
